# Isolation, screening and identification of ethanol producing yeasts from Ethiopian fermented beverages

**DOI:** 10.1016/j.btre.2023.e00815

**Published:** 2023-10-04

**Authors:** Dagnew Bitew, Anteneh Tesfaye, Berhanu Andualem

**Affiliations:** a Department of Biology, Mizan-Tepi University, P. BOX: 260, Ethiopia; b Institute of Biotechnology, University of Gondar, P.BOX: 196, Ethiopia; c Institute of Biotechnology, Addis Ababa University, P.BOX: 1176, Ethiopia; d BioTEI, Winnipeg, Manitoba, Canada

**Keywords:** Banana peel, Bioethanol, Biomass, *S. cerevisiae*, Renewable energy

## Abstract

The growing demand for renewable energy sources such as bioethanol is facing a lack of efficient ethanologenic microbes. This study aimed to isolate and screen ethanologenic yeasts from Ethiopian fermented beverages. A progressive screening and selection approach was employed. Selected isolates were evaluated for bioethanol production using banana peel waste as substrate. A total of 102 isolates were obtained. Sixteen isolates were selected based on their tolerance to stress conditions and carbohydrate fermentation and assimilation capacity. Most found moderately tolerant to 10 %, but slightly tolerant at 15 and 20 % (v/v) ethanol concentration. They yield 15.3 to 20.1 g/L and 9.1 ± 0.6 to 12.9 ± 1.3 g/L ethanol from 2 % (w/v) glucose and 80 g/L banana peel, respectively. Molecular characterization identified them as *Saccharomyces cerevisiae* strains. Results demonstrate insight about their potential role in the ethanol industry. Optimization of the fermentation conditions is recommended.

## Highlights

• Of 102 isolates, 16 potential ethanologenic isolates were selected.
• They produce 15.3 to 20.1 g/l ethanol from 2 % (w/v) glucose.
• They yield 9.1 to 12.9 g/L ethanol from 80 g of banana peel.
• Genotyping using internal transcribed spacer (ITS) regions identified them as *S. cerevisiae* strains.

## 1 Introduction

Energy is a pacemaker for mankind's activity and a nation's development [1]. Global energy demand has been entirely dependent on fossil fuels for centuries [2]. The growing global population and industrialization led to the rapid depletion of fossil fuels, which in turn created a global energy crisis. The annual global crude oil production is projected to decline to 5 billion barrels in 2050 [3]. On the other hand, fossil fuels, unevenly distributed all over the world, are responsible for the emission of greenhouse gases into the common environment that everybody shares [4,5]. Nowadays, the problem of climate change is a phenomenon that we are seeing and feeling in terms of devastating events [6]. As a result of the energy crisis and climate change, which are currently troubling the world, clean and safe alternative energy supplies have become the concern of all countries [7,8].

Bioethanol can be produced from sugar and/or starch-based energy crops and lignocellulosic biomass [9]. Bioethanol can replace fossil fuels partially or fully [10]. It is recognized as an important transportation fuel as a result of its high octane number, oxygen content (35 %), emissions of less greenhouse gases [11], blendability with gasoline and petrol [11,12], and can be produced from diversified lignocellulosic materials [13]. Indeed, it is biodegradable, less toxic, and ideal for meeting the energy demands of fossil-fuel poor countries [14]. Thus, bioethanol production is growing from 13.2 billion liters in 2000 [15] to 109.8 billion liters in 2019 [16]. However, bioethanol production is hampered by numerous factors such as sustainability of substrates, biomass recalcitrance [17], lack of pretreatment methods to efficiently release fermentable sugars [18,19], co-hydrolysis of inhibitor substances [20,21], and poor performance of fermentative microbes [19].

Global bio-energy production is principled on the exploitation of viable and cheap substrates to produce high-quality energy products [22]. Waste biomass is critically important since it is cheap, abundantly found, and sustainably available [23]. Utilizing of such substrates avoids the conflict between whether edible materials are for human consumption or industrial purposes [24]. Fruit waste is considered an attractive biomass for bioethanol production since it has a high amount of fermentable sugars, cellulose and hemicellulose with low lignin content [25,26]. In particular banana fruit waste contains significant amount of fermentable sugar and investigated as an important substrate for the production of high quality bioethanol [27], [28], [29]. These wastes are abundantly found in fruit markets, juice vendors, and juice processing industries and impose environmental problems. Hence, generating energy from it while cleaning the environment is like hitting two birds with one stone [30].

One of the challenges in large-scale bioethanol production is obtaining efficient microorganisms that are able to ferment a variety of sugars released during hydrolysis while also tolerating stress conditions [31,32]. Yeasts are known and preferred over bacteria for ethanol production as a result of their good fermentation capacity and tolerance to ethanol, low pH, and other fermentation byproducts. *Saccharomyces cerevisiae*, in particular, is a workhorse microbe that has been used by industries to produce bioethanol from lignocellulosic biomasses [33,34]. It has gained support due to its tolerance to a wide range of stress conditions, ethanologenic characteristics, and good fermentation performance [35,36]. However, commercialized ethanologenic yeasts have faced criticism due to their susceptibility to thermophilic conditions, co-hydrolyzed inhibitory chemicals, and inability to ferment pentose sugars, which make up a significant portion of fermentable sugars generated from lignocellulosic biomass [37,38]. Hence, there is a need for new ethanologenic microbes as bioethanol production advances [36,38].

Fermented food and beverages are considered an important source of ethanologenic yeasts that are able to tolerate a wide range of stress conditions, such as nutrient starvation and complexity, low pH, temperature fluctuations, and high osmotic stress [39], [40], [41]. Therefore, this study was designed to isolate ethanologenic yeast from Ethiopian traditional fermented beverages, evaluate their ethanol productivity using banana peel as a substrate, and identify the best-performing isolates for bioethanol industrial applications.

## 2 Materials and methods

### 2.1 Sample collection and isolation of yeasts

Samples of traditional fermented beverages, i.e. *tella, tej, korefe, areki tinsese, terahi, bubegne, shamita, borde*, and *cheka* were collected from different sites in Amhara Regional State and Arba Minch town, Gamo Gofa Zone, South Nations Nationalities and People's Regional State, and transported to the Cellular and Microbial Laboratory, Institute of Biotechnology, University of Gondar with an ice box to avoid the dynamics of microbes in the sample. All samples were kept at 4 °C until processed [42].

All collected fermented beverages were serially diluted (10^−1^ to 10^−5^) using peptone water as diluents. Twenty microliters (20 µL) of sample from 10^−4^ and 10^−5^ dilution factors in each beverage were spread on yeast extract peptone dextrose agar (YPDA) (containing yeast extract, 10 g/L; peptone, 10 g/L; D-glucose, 20 g/L, and 1 L sterile distilled water, 50 µg chloramphenicol/mL and adjusted to pH 5 before autoclaving). Inoculated agar plates were incubated at 30 °C for 72 hrs. Representative colonies that showed comparatively different cultural characteristics were sub-cultured onto different YDPA plates and incubated at 30 °C for 72 hrs [43].

### 2.2 Morphological characterization, designation and preservation of yeast isolates

A separately grown colonies of each isolate were examined for cultural characteristics (colony shape, margin, elevation, size, color and surface texture). Once characterized, yeast isolates were designated based on the site and type of fermented beverage they were isolated from. Then each isolate was preserved in malt extract agar slants (40 g/L) using test tubes and in vials with trypetone soya broth containing 20 % glycerol (v/v) for further use [44].

### 2.3 Physiological and biochemical characterization of isolates

#### 2.3.1 Pre-selection of yeast isolates

Purified yeast isolates were screened for glucose fermentation performance according to [45]. A 1 mL of 24 hrs old culture of each yeast isolate (adjusted to 10^8^ CFU/mL) was inoculated to 40 mL YEPD broth (yeast extract 10 g/L, peptone 10 g/L, glucose 20 g/L and 1 L distilled water, 4 µg/L phenol red adjusted to pH 5)) in test tubes containing a 5 mL size inverted Durham tube. Inoculated tubes were incubated at 30 °C for 48 hrs. Gas formation was checked every 12 hrs and isolates were selected based on the volume of gas in the Durham tube after 48 hrs of incubation [46].

#### 2.3.2 pH requirement evaluation

A protocol developed by [47] was used to examine the pH tolerance and requirement of isolates in terms of their glucose fermentation and growth at YPD broth adjusted to different pH values (3, 3.5, 4, 4.5, 5, 5.5, 6 and 6.5). A 1 mL of each yeast isolate (10^8^ CFU/mL, adjusted by 0.5 McFarland standard) was inoculated separately into 40 mL YEPD broth medium in bottles and test tubes and incubated at 30 °C. Fermentation performance response was estimated by measuring the formation of gas (in terms of the amount of liquid replaced by gas) in a 5 mL Durham tube after 48 hrs of incubation. The formation of gas was checked every 12 hrs. The growth response of isolates was evaluated *via* variation in their dry biomass at different pH values after 72 hrs.

#### 2.3.3 Temperature requirement evaluation

The yeast isolates’ fermentation performance and growth response at different incubation temperatures were examined. A 1 mL of each yeast isolate (10^8^ CFU/mL) was inoculated separately into 40 mL YEPD broth (adjusted to pH 4.5) in bottles and test tubes and incubated at different temperatures. Growth response was evaluated *via* variation in their dry biomass, and fermentation response was estimated by measuring the formation of gas (in terms of the amount of liquid replaced by gas) in the Durham tube. The formation of gas was evaluated every 12 hrs [47].

#### 2.3.4 Carbohydrate fermentation

Carbohydrate molecules such as glucose, sucrose, maltose, galactose, fructose, lactose, mannose, arabinose and xylose were used for the carbohydrate fermentation test. This test was conducted according to [48] with slight modification. A 1 mL of 24 hrs old culture of each yeast isolate (adjusted to 10^8^ CFU/mL) was inoculated to 40 mL YPD broth medium (yeast extract 10 g/L, peptone 10 g/L, carbohydrate molecules 20 g/L and 1 L distilled water; 4 µg/L phenol red adjusted to pH 4.5) in test tube and incubated at 30 °C for 72 hrs. The formation of gas was checked every 12 hrs. The fermentation of each carbohydrate molecule by each yeast isolate was estimated *via* observing the formation of gas in the Durham tube with or without medium color change after 72 hrs of incubation [49].

#### 2.3.5 Carbohydrate assimilation test

A modified auxanographic method developed by [50] was used to evaluate the carbohydrate assimilation test. A modified yeast nitrogen base (YNB) medium (containing ammonium sulfate 5 g/L, potassium phosphate monobasic 0.85 g/L, potassium phosphate dibasic 0.15 g/L, magnesium sulfate 0.5 g/L, sodium chloride 0.1 g/L, calcium chloride 0.1 g/L, yeast extract (fermentable carbohydrate free) 1 g/L and Agar 15 g/L) was used to check the isolate's carbohydrates assimilation spectrum. A modified YNB medium containing 2 % (w/w/) carbohydrate molecules (glucose, sucrose, maltose, lactose, fructose, galactose, mannose, arabinose, mannitol, starch and xylose) was sterilized and poured at 4 mm thickness aseptically onto 90 mm Petri plates and allowed to solidify. Then the dried medium was inoculated with 10 µL of a 24 hrs old culture of each yeast isolate (adjusted to 12 × 10^8^ CFU/mL) and incubated at 30 °C and examined for growth every 2 days for up to 5 days. A carbohydrate free medium was used as a control. Assimilation was considered positive if there was considerable growth in the plate containing the carbohydrate as compared with the control and negative if there was no growth.

#### 2.3.6 Osmotolerance evaluation

A 5 mL YPD broth medium containing (60 %, 70 % and 80 % (w/w) of glucose in a separate flask and adjusted to pH 4.5), was dispensed into screw-cap test tubes and sterilized. Each test tube was inoculated with 1 mL of each yeast isolate (standardized at 10^8^ CFU/mL) and incubated at 30 °C for 72 hrs. The growth of each yeast isolate in each glucose concentration was determined qualitatively by observing its turbidity and quantitatively by measuring the dry mass [51,52].

#### 2.3.7 Ethanol tolerance test

Ethanol tolerance of isolates was examined in terms of growth, fermentation performance response, and survival percentage in YPD broth containing different concentrations (10 %, 15 % and 20 % (v/v)) of absolute ethanol. A 40 mL YPD broth medium containing those concentrations of absolute ethanol separately and adjusted to pH 4.5, was dispensed into screw-cap test tube and a 300 mL glass bottle for fermentation and growth response respectively. Each test tube was inoculated with 1 mL of each yeast isolate (standardized at 10^8^ CFU/mL) separately and incubated at 30 °C for 72 hrs. The amount of gas formed was considered to evaluate isolates’ fermentation performance and indirectly their tolerance to each ethanol concentration tested. On the other hand, growth was determined by measuring the dry mass after 72 hrs of incubation [41].

Similarly, 1 mL (10^8^ CFU/mL) of each isolate was inoculated into 40 mL of YPD broth containing different concentrations of ethanol as mentioned above and incubated at 30 °C. Then, after 72 hrs of incubation, the culture broth of each isolate was diluted (10^−1^ to 10^−3^) with phosphate buffer saline, and a 20 µL sample from the 10^−3^ dilution was spread on YPDA plate and incubated at 30 °C for 72 hrs. The survival percentage of each isolate was estimated by comparing it with its counterpart grown in pure YPD broth and incubated at 30 °C for 72 hrs. The survival percentage was calculated as Eq. (1):

(1) $$\begin{array}{c} \mathrm{Survival}\;\mathrm{Percentage}=\frac{\mathrm{Total}\;\mathrm{cell}\;\mathrm{count}\;\mathrm{after}\;\mathrm{stressed}\;}{\mathrm{Total}\;\mathrm{cell}\;\mathrm{count}\;\mathrm{of}\;\mathrm{unstressed}/\mathrm{control}}\;X100 \end{array}$$

Ethanol tolerance was determined based on the percentage of survival: highly tolerant (>50 % survival), moderately tolerant (25–50 % survival), and slightly tolerant (<25 % survival) [53].

#### 2.3.8 Evaluation of ethanol productivity

Yeast isolates that withstand the above mentioned stressful environment and aggressively ferment and assimilate tested carbohydrate molecules were selected. These isolates were further screened for ethanol production using yeast extract broth containing 2 % glucose. A 1 mL of 24 hrs old cultures of each isolate (adjusted to 12 × 10^8^ CFU/mL) was inoculated into a 300 mL of broth (pH 4.5). The inoculated bottles were sealed with parafilm to create anaerobic condition and incubated at 30 °C for 72 hrs under shaking condition at150 rpm. Then, the produced ethanol was separated from the aqueous solution at 78.4 °C using fractional distillation. The amount of ethanol produced was measured colorimetrically using the potassium dichromate method [54]. Unassimilated glucose was estimated using the 3, 5- dinitrosalicylic acid (DNS) method, according to [55]. Ethanol and glucose standard curves were constructed using absolute ethanol and glucose, analytic grade quality.

### 2.4 Flocculation property of isolates

The flocculence of isolates were checked at 24, 48 and 72 hrs according to [56] with little modification. A 1 mL of 24 hrs culture of each isolate (10^8^ CFU/mL) was inoculated separately into 40 mL of YPD broth and incubated at 30 °C. To determine cells in suspension, inoculated broth were serially diluted (10^−1^ to 10^−3^) after 24, 48 and 72 hrs of incubation. Then 20 µL from the 10^−3^ dilution factor was spread onto YPDA plate and incubated at 30 °C for 72 hrs.

Total flocculated cells were determined after overnight cold induced lagering at the end of fermentation. Yeast cell mass was harvested by centrifugation 16,000 rpm for 10 min, washed three times with physiological saline and 1 g of washed cell mass of each strain was suspended in 40 mL of saline and serially diluted (10^−1^ to 10^−3^). A 20 µL from the 10^−3^ dilution factor was spread plated and incubated at 30 °C for 72 hrs. Total colonies were counted in both cases and flocculation percentage of each isolate was determined by the following formula [57].

(2) $$\begin{array}{c} \mathrm{Floculation}\;\mathrm{Percentage}=\frac{\mathrm{Total}\;\mathrm{cell}\;\mathrm{count}\;\mathrm{BL}-\;\mathrm{Total}\;\mathrm{cell}\;\mathrm{count}\;\mathrm{AL}}{\mathrm{Total}\;\mathrm{cell}\;\mathrm{count}\;\mathrm{BL}}\;X\;100 \end{array}$$

Where, BL-Before laagering and AL-After laagering

### 2.5 Evaluation bioethanol production potential of selected isolates

#### 2.5.1 Banana peel collection and processing

Banana peel waste was collected from fruit selling areas in Azezo district, Gondar, Ethiopia. Collected peel waste was rinsed with tap water to clean soil and associated impurities and sun dried. Following, the dried peel waste was oven-dried at 60 °C for 10 min and ground with a grinder. The obtained powder waste was sieved, and the fine powder was subjected to steam pretreatment [29].

#### 2.5.2 Pretreatment and hydrolysis

Banana peel powder was steam pretreated and acid hydrolyzed according to [1]. Eighty gram of peel powder was suspended in 1 L of distilled water in screw caped bottles and autoclaved at 121 °C and 15 psi pressure for 30 min. Then, pretreated peel powder was acid hydrolyzed with 1.5 % (v/v) sulfuric acid and heated at 91 °C for 21 min. The obtained hydrolysate was filtered with Whatman Grade 1 filter paper (11 μm) and adjusted to pH 4.5 (optimum pH) with 1 M NaOH.

#### 2.5.3 Banana peel chemical characterization

The pretreated banana peel sample was dried in hot air oven at 72 °C. The dried sample was then pulverized to a particle size smaller than 1 mm in a mechanical grinder [58]. Banana peel cellulose, hemicellulose, soluble lignin and pectin content was analyzed following the protocols of [59]. Total sugar and reduced sugar amounts were quantified by the sulfuric phenol method and the 3–5 dinitrosalicylic acid (DNS) method according to [18,55], respectively. The ash content was determined by the Association of Official Analytical Chemists (AOAC) method 2000 [60]. This assay was conducted in triplicate.

#### 2.5.4 Fermentation and determination of ethanol content

A batch fermentation system was employed, and the obtained hydrolysate was directly used for fermentation. Fermentation was done in 300 mL capped glass bottles containing 250 mL hydrolysate. Each bottle was inoculated with a 2 % (v/v) 24 hrs old culture of each isolate (10^8^ CFU/mL initial cell density). Inoculated fermentation bottles were sealed with parafilm (Bemis, USA) to ensure anaerobic condition, and incubated in CO_2_ incubator at 30 °C for 72 hrs under shacking condition at 150 rpm [61]. The supernatant was separated from yeast cell mass and insoluble solid mater *via* centrifugation at 4000 rpm for 10 min. Then the produced ethanol was separated from the aqueous solution at 78.4 °C using fractional distillation, and its amount was estimated colorimetrically using the potassium dichromate method [54]. Ethanol standard curve was constructed using standard solutions of absolute ethanol [62]. Unfermented residual sugar was estimated using the 3, 5- dinitrosalicylic acid (DNS) method, according to [55]. Fermentation parameters such as sugar utilization percentage, fermentation efficiency, ethanol yield, and ethanol productivity were calculated according to [63,64].

(3) $$\begin{array}{c} \mathrm{Sugar}\;\mathrm{util}\mathrm{izat}\mathrm{ion}\%\;=\;\frac{\;S1-S2}{S1}X100 \end{array}$$

Where S1 is the initial sugar concentration in the hydrolysate and S2 is the unconsumed residual sugar concentration in the fermented broth.

(4) $$\begin{array}{c} \mathrm{Ferm}\mathrm{enta}\mathrm{tion}\;\mathrm{effi}\mathrm{cien}\mathrm{cy}\;=\;\frac{\;\mathrm{Practical}\;\mathrm{Yield}}{\mathrm{Theortical}\;\mathrm{Yield}}X100 \end{array}$$

Where practical yield is the ethanol produced and theoretical yield is s 0.511 per gram of sugar consumed.

(5) $$\begin{array}{c} \mathrm{Prac}\mathrm{tical}\;\mathrm{etha}\mathrm{nol}\;\mathrm{yield}\;=\;\frac{\;\mathrm{Ethanol}\;\mathrm{Concentration}\;\left(g/L\right)\;\mathrm{in}\;\mathrm{fermented}\;\mathrm{broth}}{\mathrm{Sugar}\;\mathrm{Consumed}\;\left(g/l\right)} \end{array}$$

(6) $$\begin{array}{c} \mathrm{Etha}\mathrm{nol}\;\mathrm{Prod}\mathrm{ucti}\mathrm{vity}\;=\;\frac{\;\mathrm{Ethanol}\;\mathrm{Concentration}\;\left(g/L\right)\;\mathrm{in}\;\mathrm{fermented}\;\mathrm{broth}}{\mathrm{Fermentation}\;\mathrm{time}\;\left(\mathrm{hrs}\right)} \end{array}$$

(7) $$\begin{array}{c} \mathrm{Yield}\;\mathrm{of}\;\mathrm{etha}\mathrm{nol}\;\left(g/\mathrm{kg}\right)\;\mathrm{of}\;\mathrm{dry}\;\mathrm{biom}\mathrm{ass}\;=\;\frac{\;\mathrm{Ethanol}\;\mathrm{Conc}.\;\left(g/L\right)\;\mathrm{in}\;\mathrm{fermented}\;\mathrm{broth}}{\mathrm{Dry}\;\mathrm{weight}\;\mathrm{of}\;\mathrm{substrate}\;\mathrm{biomass}}x1000 \end{array}$$

The commercial strain, *S. cerevisiae* ©DB (*Sc* ©DB) obtained from Dashen Brewery Factory, Gondar, Ethiopia was used as a control. Banana peel hydrolysate fermentation and ethanol yield parameters assay were conducted in triplicate.

### 2.6 Molecular identification of ethanologenic yeasts

#### 2.6.1 DNA extraction

The genomic DNA of selected isolates was extracted using the GenEluteTM Fungal/Plant Genomic DNA Miniprep Kit (Sigma Aldrich). The concentration and quality of extracted DNA were determined using NanoDrop and gel electrophoresis and kept at -20 °C until it was needed for PCR amplification.

#### 2.6.2 Amplification of ITS region

The internal transcribed spacer regions (ITS 1 and ITS 2) of the rRNA gene were used as a barcode and amplified using universal primers ITS1F (5′-CGGGATCCGTAGGTGAACCTGCGG-3′) and ITS4R (5′-CGGGATCCTCCGCTTATTGATATGC-3′) (Sigma Company). A 20 µL containing (5 x FIREPol® Master Mix 4 μL, forward primer 0.3 μL (10 pmol/μL), reverse primer 0.3 μL (10 pmol/μL), 3 µL (30 ng), template DNA, and 13.4 µL nuclease-free water. The PCR was run for about 30 cycles with initial denaturation at 95 °C for 5 min, denaturation at 95 °C for 30 second, annealing temperature at 54 °C for 30 second, extension at 72 °C for 2 min, and final extension 72 °C for 7 min (Solis BioDyne Data Sheet). PCR products were analyzed by loading 5 μL onto 2 % agarose gels containing 3 μL ethidium bromide and visualized under UV light.

#### 2.6.3 Sequencing and phylogenetic tree construction

PCR products were purified and sequenced using standard sequencing. Sequenced data were edited using BioEdit software package and checked for similarity *via* BLAST search program (https://10blast.ncbi.nlm.nih.gov/Blast.cgi) to those previously deposited sequences from GenBank databases. Species determination was done by considering identity percentage ≥ 99 %, E- value = 0 and query coverage ≥ 95 %. Finally, sequence of each isolate was submitted at GenBank of NCBI. Then ITS sequences of isolates were aligned with the multiple alignment program CLUSTAL W and neighbor-joining method was applied to construct a phylogenetic tree using MEGA software version 11.0.

### 2.7 Statistical analysis

Experimental data were generated in triplicate and analyzed using one-way ANOVA analysis using SPSS version 23. Tukey post hoc multiple comparison test was used for mean comparison. The significant difference among variables was considered at *p* ≤ 0.05. The results are expressed as mean ± standard deviation

## 3 Results and discussions

### 3.1 Isolation, screening and progressive selection of yeast isolates for ethanol production

The shocking impacts of climate change and the lack of security in fossil fuel supply have forced countries across the world to look for environmentally friendly energy sources such as bioethanol [65]. However, its large scale high-quality production with reasonable costs is hindered by a lack of suitable ethanologenic microbes [66,67]. This urged searching for robust ethanologenic yeasts that are able to meet the demands of the bioethanol industry [19]. Thus, in the present study, a total of 102 yeast isolates were isolated from Ethiopian traditional fermented beverages: *tella, tej, korefe, areki tinsese, terahi, bubegne, shamita, borde*, and c*heka.* Colony morphological characterization revealed that all are circular shaped and most are white colored with an entire margin and a smooth texture. Morphologically distinct isolates from a given fermented beverage were considered different yeast species and/or strains and were designated differently (data not shown). This is in agreement with several reports that states traditional fermented beverages are an important niche for yeasts in general and ethanol producing yeasts in particular [47,68,69].

#### 3.1.1 Pre-selection of isolates

**Fig. 1:**
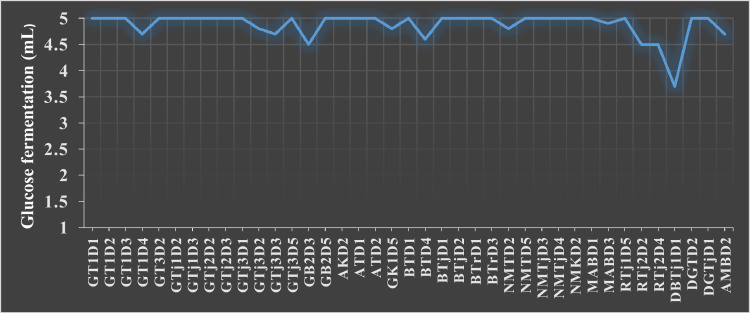
Glucose fermentation performance of pre-selected yeasts isolates.

Glucose fermentation has been regarded as a confirmatory test for ethanol production [41]. For that reason, glucose fermentation was used as a pre-selection hurdle to exclude non-fermenters and weak fermenters. Among 102 yeast isolates, 39 (38 %) formed gas that ranged between 3.7 ± 0.29 and 5 ± 0.00 mL after 48 hrs of incubation at 30 °C (Supplementary data (Sd) Table 5, Fig. 1). These were selected and subsequently screened for pH and temperature requirement and tolerance.

#### 3.1.2 pH and temperature requirement and tolerance

The bioethanol production imposes stressful conditions on ethanologenic yeasts [70]. Hence, apart from fermentation efficacy and ethanol productivity, tolerance to stressing factors such as pH and temperature are recognized as desirable features of ethanologenic yeasts [33,71,72]. Thus, extensive screening of yeast isolates for these physiological characteristics is considered as a pre-requisite so as to get an ideal ethanologenic strain to be used in industrial scale bioethanol production [73,74].

Yeast are acidophilus and they love the initial medium pH (4.5 −5.5) during ethanol production. However, the ultimate lowered pH as a result of organic acid production imposes a deleterious effect on their viability and fermentation performance [75,76]. Therefore, tolerance to low pH is an essential feature of ethanol producing yeast [77]. Pre-selected isolates were screened for their pH requirements and tolerance. They were able to grow and ferment glucose in all tested pH values, with dry weight and gas formed ranging from 52.3 ± 6.43 (at pH 6.5) to 95.7 ± 5.13 mg (at pH 4.5) and 0 (at pH 6.5) to 5.0 ± 0.0 mL (at pH 4.5), respectively. Both fermentation and the growth of isolate decreased as the acidity of the medium decreased. The maximum growth and fermentation performance were recorded at pH 4.5, suggesting that this is an optimum pH environment for these isolates (Sd Table 1 and 2). The results of this study are in line with the fact that nearly all yeasts prefer to grow and ferment in acidic conditions [78,79]. The considerable growth and fermentation potential of pre-selected isolate at low pH (3 and 3.5) revealed that they are acidophilus; and this is critically important in avoiding bacterial contamination during the ethanol fermentation process.

Temperature is the key factor for fermentation [80]. Keeping the optimum temperature during the fermentation process is a difficult task in all bioethanol plants [81]. Deviating from the ideal temperature affects the specific growth rate of yeast strains, which in turn affects ethanol production [82,83]. Hence, screening yeast isolates for their temperature requirement and tolerance is a basic component in the search and development of ethanologenic yeasts. Results indicated that the growth and fermentation responses of pre-selected yeast isolates increased as the temperature increased to 35 °C. However, their growth was sharply declined at 40 °C, while fermentation completely ceased. This is consistent with [80] and [83,84], reported that yeast growth, viability and the fermentation potential are repressed as the temperature increases above 35 °C. This decrease in viability and fermentation is attributed to the synergetic inhibitory effect of high temperature and the production of ethanol [85]. Maximum growth and fermentation performance were recorded at 30 °C. (Sd Table 3 and 4). This agrees with [79,86,87], stated that most yeasts showed better growth and fermentation performance at 30 °C.

#### 3.1.3 Carbohydrate fermentation and assimilation

Yeasts vary in their fermentation and assimilation of carbohydrate molecules. A broader carbohydrate fermentation and assimilation spectrum of ethanologenic yeast is crucial for the effective conversion of various sugars in the biomass to ethanol [88]. Results of this study showed that pre-selected isolates ferment glucose, sucrose, maltose, fructose, mannose and galactose to different extents after 48 hrs of incubation at 30 °C. Regarding their carbohydrate assimilation spectrum, they were able to grow on glucose, sucrose, maltose, galactose, fructose, and mannose. However, none of them ferment and assimilate arabinose, mannitol, lactose, xylose and starch **(**Sd Table 5 and 6**)**. This is in line with the reports of [41,42,47], investigated that most yeast isolates ferment and assimilate glucose, sucrose, maltose, fructose and galactose. In addition, [89] investigated that among 90 *Saccharomyces* species tested, the majority of strains showed a very poor metabolic index for arabinose and xylose. Carbohydrate fermentation and assimilation profiles are a species-dependent properties; any variation in carbohydrate utilization may emanate from their taxonomic variation. According to their carbohydrate fermentation results, 30 better-performing isolates were selected among 39 pre-selected isolates and further screened. The carbohydrate fermentation and assimilation pattern of the investigated isolates and the reference strain, *Sc* ©DB, were the same. This might be due to they are belongs to the same taxonomic group.

#### 3.1.4 Osmotic and ethanol tolerance evaluation of isolate

Ethanologenic yeasts always face osmotic stress as a result of high sugar and solute concentrations, particularly during high-gravity fermentation. Later, the accumulation of ethanol further imposes stress and affects yeast viability and vitality [89]. In high-gravity fermentation, a high ethanol yield is expected, however, the hyperosmotic condition imposes a deleterious effect on yeast proliferation and viability. This eventually decreases sugar to ethanol conversion efficiency and the final ethanol titer [72]. Hence, prevailing under high osmotic pressure and ethanol concentration are other important features of ethanologenic yeasts, particularly in the case of high-gravity bioethanol fermentation [90], [91], [92]. Thus, screening yeast isolates for tolerance to the aforementioned stress factors is very critical to get efficient ethanologenic strains. Pre-selected isolates were able to grow at each glucose concentration with dry weight (mg) ranging from 3.7 ± 0.58 to 8.2 ± 0.68, 1.8 ± 0.29 to 4.7 ± 0.58 and 0.9 ± 0.10 to 2.7 ± 0.28 respectively (Sd Table 7). This agrees with [51], reported yeast strains that are able to grow in sugar concentrations up to 80 % (w/w) sugar. Since osmotic tolerance is a species-dependent feature, screening of isolates at elevated concentrations makes the selection of tolerant isolates easier. The tolerance of these isolates to high sugar concentration showed that they are a potential candidate for high-gravity industrial production of bioethanol.

Ethanol tolerance assessment revealed that among 16 selected isolates 5 were highly tolerate, and 10 were moderately tolerant and 1 was slightly tolerant to 10 % (v/v) ethanol concentration with survival percentages ranging from 21.7 ± 5.0 to 65.6 ± 6.9. But they were slightly tolerant at 15 % and 20 % (v/v) ethanol concentration with an insignificant survival rate **(**Table 1**).** Ethanol is toxic to the cell, and a reduction in growth, fermentation performance, and survival percentage is expected when ethanol concentration increases. The findings of this study are consistent with [41,83], who found that *S. cerevisiae* isolates survived better at 10 % ethanol concentrations while their growth declined when the ethanol concentration increased beyond 12 %. This is also in line with [93], reported that about 86 %, 35 %, and 17 % of isolates grow well in yeast malt extract medium (YM) containing 10 %, 12 %, and 14 % (v/v) ethanol respectively. Differently, the study of [41] stated that seventeen isolates, isolated from bio-wastes and co-products of sugar factories were highly tolerant to16 % ethanol concentration and one was exceptionally tolerant to 20 % ethanol. The discrepancy in ethanol tolerance might be due to variation in species and/or strains, genetic makeup and sources they are isolated from.

**Table 1:** Ethanol tolerance and survival percentage of selected yeast isolates.

Isolate	Tolerance to different ethanol concentrations						Survival percentage of isolates		
	Glucose fermentation (mL)			Growth (in terms of dry weight in mg)					
	10	15	20	10	15	20	10	15	20
GT1D3	2 ± 0.00^b^	-	-	81.0 ± 3.00^b^	-	–	41.6 ± 8.5^c^	6.1 ± 3.9^b^	1.6 ± 1.4^b^
GT1D4	2 ± 0.43^b^	-	-	75.3 ± 4.04^b^	-	–	50.3 ± 2.8^d^	5.9 ± 3.4^b^	–
GT3D2	2.5 ± 0.50^b^	-	-	77.0 ± 2.65^b^	-	–	57.1 ± 4.3^f^	9 ± 0.8^b^	3.2 ± 1.1^c^
GTj1D3	1.8 ± 0.25^b^	-	-	79.3 ± 3.51^b^	-	–	50.5 ± 7.4^d^	7.4 ± 2.1b	–
GTj2D2	1.9 ± 0.96^b^	-	-	75.3 ± 3.21^b^	-	–	65.6 ± 6.9^e^	11.5 ± 6.9^c^	2.8 ± 1.0^c^
AKD2	3.5 ± 0.50^c^	-	-	81.3 ± 2.51^b^	-	–	56.5 ± 11.0^d^	5 ± 2.1^b^	0.3 ± 0.6^a^
ATD2	3.4 ± 0.36^c^	-	-	78.0 ± 1.73^b^	-	–	40.7 ± 8.3^c^	4.2 ± 0.9^a^	–
GK1D5	2.3 ± 1.15^b^	-	-	76.3 ± 1.53^b^	-	–	57.7 ± 1.8^d^	8.7 ± 4.0^b^	1.8 ± 0.4^b^
BTD1	4.0 ± 0.50^c^	-	-	76.7 ± 3.78^b^	-	–	56.6 ± 11.0^d^	-	–
BTrD1	2 ± 0.50^b^	-	-	78.0 ± 3.00^b^	-	–	31.2 ± 11.3^b^	9.5 ± 0.3^b^	–
NMTD5	2.6 ± 0.51^b^	-	-	78.0 ± 2.64^b^	-	–	21.7 ± 5.0^a^	-	–
NMTjD4	2.3 ± 0.26^b^	-	-	76.3 ± 2.5^b^	-	–	27.3 ± 6.1^b^	-	–
MABD1	2.1 ± 0.17^b^	-	-	79.0 ± 1.73^b^	-	–	37.8 ± 8.3^c^	4.7 ± 2.8^a^	–
RTj2D2	2.8 ± 1.10^c^	-	-	75.7 ± 5.13^b^	-	–	29.1 ± 5.7^b^	-	–
DGTjD1	3.5 ± 0.25^c^	-	-	76.7 ± 6.11^b^	-	–	31.9 ± 10.8^b^	8 ± 6.4^b^	–
AMBD2	3.3 ± 0.26^c^	-	-	76.0 ± 4.00^b^	-	–	39.2 ± 5.5^c^	4.5 ± 2.7^a^	–
*Sc*©DB	1.6 ± 0.40^b^	–	–	68.7 ± 4.72^b^	–	–	33.3 ± 5.5^b^	–	

**Key**: Mean values superscripted with different letters across the column are significantly different at *P*-value ≤ 0.05. (-) denotes no growth, fermentation and survival.

#### 3.1.5 Ethanol productivity screening

**Fig. 2:**
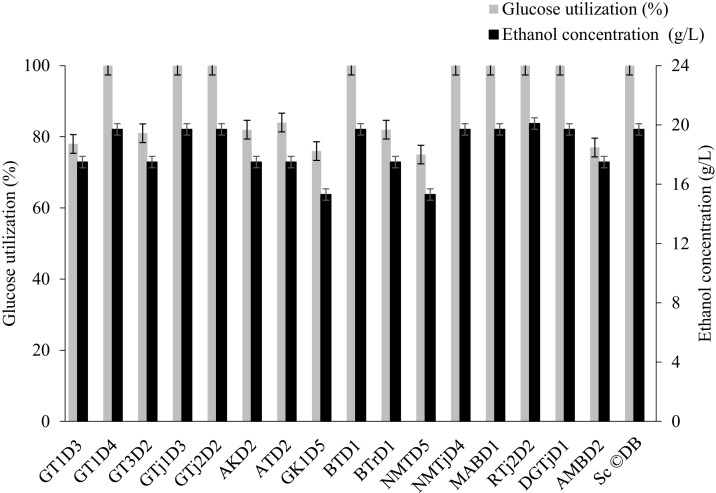
Ethanol production and glucose consumption performance of selected isolates.

Screening for ethanol production demonstrated that all selected isolates produce a considerable amount of ethanol with significant glucose consumption. The obtained ethanol yield ranged from 15.3 to 20.1 g/L, with the degree of glucose consumption varying between 73 and 100 % after 72 hrs of incubation at 30 °C (Fig. 2). The results are in line with the findings of [94], reported ethanol production ranging from 1.09 % (10.9 g/L) to 2.49 % (24.9 g/L) using YPD medium containing 2 % (w/w) glucose. [95] also reported that ethanol production ranged from 12.99 ± 0.37 to 23.10 ± 0.25 g/L and 13.83 ± 0.34 to 25.94 ± 0.52 g/L by osmotolerant *S. cerevisiae* strains using YPD broth containing 5.5 % glucose and prepared by reverse osmosis water and sea water, respectively.

#### 3.1.6 Flocculation property of selected isolates

**Fig. 3:**
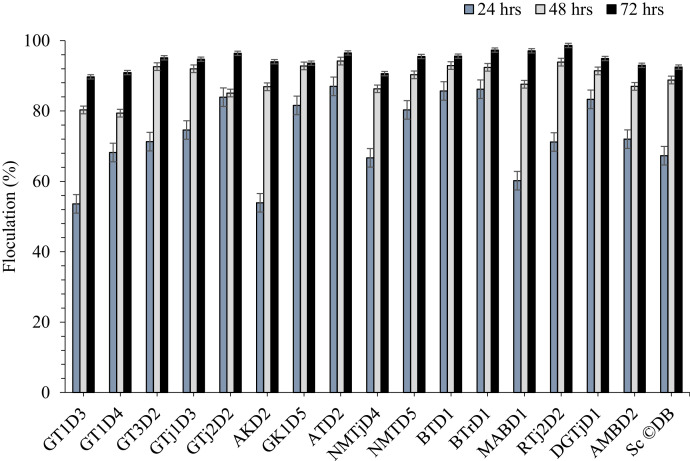
Flocculation percentage of isolate after 24, 48 and 72 hrs of incubation.

Flocculence is another desired trait of ethanologenic yeasts [96]. It is a spontaneous, reversible aggregation of single yeast cells into clumps and subsequent sedimentation to the conical bottom of the fermenter. This makes it easier to clarify yeasts from the medium at the end of bulk fermentation and prepare for repitching. The timing of flocculation is critically important, and it should be neither too early nor too late [97]. The early flocculating yeasts tend to settle and clump together very quickly and fail to complete the fermentation of fermentable sugar, which then results in low-quality bioethanol. Results of the present study showed that all selected yeast isolates are bottom fermenters, with flocculation percentages ranging from 53.6 ± 9.4 to 89.7 ± 1.5 %, 77.7 ± 1.5 to 94.2 ± 2.8 % and 89.7 ± 4.0 to 98.6 ± 0.9 % after 24 hrs, 48 hrs and 72 hrs incubation, respectively. Their flocculence increased as time went on until the end of fermentation (Fig. 3). The results of the present study were higher than the flocculation percentage of transgenic wine yeast strains BM45-F5A and VIN13-F5A, which demonstrated 72.1 ± 3.9 % and 59.4 ± 2.7 % flocculation after 48 h of incubation at 30 °C [98]. The variance in flocculation percentage might be attributed to differences in genetic makeup, particularly variation in flocculin-encoding FLO genes, cell wall composition [99], and the physicochemical environment [100].

### 3.2 Bioethanol production from banana peel

#### 3.2.1 Banana peel characterization

As shown in Table 2 the chemical composition revealed that banana peel waste is an important source of cellulose, hemicellulose, lignin and total and reduced sugars that would be converted in to bioethanol. The cellulose, hemicellulose, lignin, pectin and ash content detected in this study agree with previous reports [1,27]. However, lower with the reports of [58]. The amount of total sugar obtained in this study is also lowered as compared with [25]. This might be attributed to variation in banana variety and sccharification approach employed. The lowered amount of cellulose, hemicellulose and lignin can be due to their transformation into monomers, which in turn related to the effectiveness of pretreatment and hydrolysis approach. The ash content is related to mineral compositions. Studies reported that the ash content of banana peel is ranged between 6.4 to 12 % for different banana variety [60,101]. The investigated ash content is within the reported range.

**Table 2:** The chemical composition banana peel powder.

Composition	Amount ( % dry matter)
Cellulose	10.5 ± 1.2
Hemicellulose	8.2 ± 2.1
Lignin	4.6 ± 1.4
Total sugar	57.2 ± 5.7
Reduced sugar	46.2 ± 1.5
Pectin	5.2 ± 2.6
Ash	6.8 ± 2.3
Other solids	7.5 ± 1.2

#### 3.2.2 Sugar utilization and ethanol production efficiency

Banana peel waste is rich in fermentable sugar and recognized as an important feedstock for the production of bioethanol [102]. However, the amount of bioethanol produced depends on the amount of fermentable sugar extracted, which in turn depends on the type of sccharification method employed [27]. Acid hydrolysis is much faster than enzymatic hydrolysis, and greater than 90 % fermentable sugar yield can be achieved with concentrated acid hydrolysis with fewer toxic degradation products than with dilute acid hydrolysis [103].

In this study, 0.57 g/g of dry banana peel powder equivalent to 46.2 ± 1.5 g/L of reduced sugar, was liberated from 80 g banana peel powder after steam pretreatment and acid treatment with 1.5 % (v/v) sulfuric acid at 90 °C for 20 min. This is low as compared with [104] obtained 45.088 g/L reduced sugar from 20 g of banana peel powder pretreated and hydrolyzed under the same conditions. It is also lowered from [105], who achieved 77.03 g/L of reduced sugar after acid hydrolyzing 50 g of matooke variety banana peel powder at 1.5 (v/v) sulfuric acid, 70 °C, and 40 min. The variation in the amount of reduced sugar extracted might be due to variations in banana peel sugar content and sccharification conditions. [106] stated that the sugar content of banana fruit varies with banana variety, growth, and environmental conditions.

The hydrolysate was fermented in batch condition. The obtained ethanol concentration ranged from 9.1 ± 0.6 to 17.2 ± 1.7 g/L, the lowest and the highest were recorded from GT3D2 and *Sc* ©DB, respectively. The sugar to ethanol conversion efficiency varied between 54.8 ± 3.9 to 90.0 ± 5.2 % with ethanol yield (YE) 0.3 ± 0.0 and 0.5 ± 0.0 g/L and productivity ranging from 0.1 ± 0.0 to 0.2 ± 0.0 g/L/h. The ethanol recovery from a kilogram of dry banana peel powder was ranged from 113.8 ± 6.9 to 214.9 ± 21.3 g/kg (Table 3). This was in agreement with the study of [27] where the reported ethanol concentration ranged between 6.7 and 26.0 g/l obtained from enzyme-hydrolyzed banana peel. It is also consistent with [107] reported 1.05 % (10.5 g/L), 1.52 % (15.2 g/L), and 1.70 % (17.0 g/L), respectively, obtained from Raja banana peel, Agung banana peel, and Nangka banana peel. But it is low as compared with [108], reported 13 g/L and 11 g/L were achieved within 10–12 hrs in simultaneous sccharification and fermentation using *S. cerevisiae* and Kluyveromyces marxianus at 35 and 41 °C, respectively, from 10 % (w/w) banana peel powder. This might be due to the isolates fermentation and assimilation capacity of a variety of carbohydrate molecules released during hydrolysis. Moreover, there can also be variations in the sugar content of banana variety peels and cell sensitivity to inhibitory compounds produced during acid hydrolysis. The recorded ethanol yield parameters of the reference strain were significantly different from the investigated isolates. This might be due to variation in tolerance of inhibitory substance co-produced during hydrolysis.

**Table 3:** Sugar utilization and ethanol yield parameters of selected isolates.

Isolate	RS (g/L)	CS (g/L)	SU%	EC (g/L)	YE (g/L)	FE (%)	EP (g/L/h)	YoE (g/Kg)
GT1D3	14.0 ± 0.5^a^	32±0.5^b^	69.6 ± 1^c^	10.2 ± 0.5^d^	0.3 ± 0.0^e^	62±3.0^h^	0.1 ± 0.0^f^	127.8 ± 5.8^b^
GT1D4	13.6 ± 0.3^a^	32.4 ± 0.4^b^	70.5 ± 0.8^c^	11.4 ± 2.1^d^	0.4 ± 0.1^e^	69.2 ± 13.0^f^	0.2 ± 0.0 g	142.8 ± 26.1^c^
GT3 D2	13.9 ± 0.1^a^	32.1 ± 0.1^b^	69.8 ± 0.3^c^	9.1 ± 0.6^d^	0.3 ± 0.0^e^	54.8 ± 3.9^e^	0.1 ± 0.0^f^	113.8 ± 6.9^a^
GTj1D3	13.9 ± 0.4^a^	32.1 ± 0.4^b^	69.8 ± 0.9^c^	10.3 ± 1.8^d^	0.3 ± 0.1^e^	62.7 ± 10.4^h^	0.1 ± 0.0 g	128.3 ± 22.4^b^
GTj2D2	13.9 ± 0.3^a^	32±0.3^b^	69.7 ± 0.6^c^	11.4 ± 1.4^d^	0.4 ± 0.0^e^	69.3 ± 9.0^f^	0.2 ± 0.0 g	141.9 ± 17.3^c^
ATD2	14±0.4^a^	32±0.4^b^	70.4 ± 0.8^c^	9.7 ± 1.1^d^	0.3 ± 0.0^e^	59.5 ± 6.9^h^	0.1 ± 0.0^f^	121.5 ± 13.2^b^
AKD2	14±0.2^a^	32±0.2^b^	69.6 ± 0.4^c^	11±1.2^d^	0.3 ± 0.0^e^	64.9 ± 10.7^h^	0.2 ± 0.0 g	137.1 ± 15.5^c^
GK1D5	13.7 ± 0.2^a^	32±0.2^b^	70.1 ± 0.5^c^	11.6 ± 1.0^d^	0.4 ± 0.0^e^	70.6 ± 6.8^f^	0.2 ± 0.0 g	144.8 ± 12.4^c^
NMTD5	14±0.4^a^	32±0.4^b^	69.7 ± 1.0^c^	10.6 ± 2.3^d^	0.3 ± 0.1^e^	64.6 ± 14.1^h^	0.1 ± 0.0^f^	132.5 ± 29.3^c^
NMTjD4	13.8 ± 0.2^a^	32.2 ± 0.2^b^	70±0.5^c^	11.5 ± 0.9^d^	0.3 ± 0.0^e^	69.2 ± 6.2^f^	0.2 ± 0.0 g	143.2 ± 10.9^c^
BTD1	13.8 ± 0.4^a^	32.2 ± 0.4^b^	70±0.9^c^	11.8 ± 1.6^d^	0.4 ± 0.1^e^	71.7 ± 11.1^f^	0.2 ± 0.0 g	147±20.3^c^
BTrD1	13.7 ± 0.2^a^	32.2 ± 0.3^b^	70.1 ± 0.5^c^	11.1 ± 1.4^d^	0.3 ± 0.0^e^	67.2 ± 8.2^f^	0.2 ± 0.0 g	138.7 ± 17.9^c^
MABD1	13.8 ± 0.3^a^	32.2 ± 0.2^b^	70±0.6^c^	11.6 ± 2.5^d^	0.4 ± 0.1^e^	70.4 ± 15.3^f^	0.2 ± 0.0 g	145±30.9^c^
RTj2D2	13.8 ± 0.3^a^	32.2 ± 0.3^b^	70.1 ± 0.7^c^	12.7 ± 0.5^d^	0.4 ± 0.0^e^	75.7 ± 6.1 g	0.2 ± 0.0 g	159±6.8^d^
DGTjD1	13.9 ± 0.4^a^	32.1 ± 0.4^b^	69.8 ± 1.0^c^	11±2.0^d^	0.3 ± 0.0^e^	67.2 ± 12.7^f^	0.2 ± 0.0 g	138.4 ± 25.0^c^
AMBD1	13.6 ± 0.4^a^	32.4 ± 0.4^b^	70.5 ± 0.9^c^	12.9 ± 1.3^d^	0.4 ± 0.0^e^	77.6 ± 6.0 g	0.2 ± 0.0 g	161.4 ± 15.8^d^
*Sc* ©DB	8.8 ± 1.8^b^	37.2 ± 1.8^a^	80.8 ± 4.0^d^	17.2 ± 1.7^f^	0.5 ± 0.0^e^	90.0 ± 5.2^k^	0.2 ± 0.0 g	214.9 ± 21.3^f^

**Key**: Mean values superscripted with different letters across the column are significantly different at *P*-value 0.05. RS (Residual Sugar), CS (Consumed Sugar), SU% (Sugar Utilization Percentage), EC (Ethanol Concentration), YE (Ethanol Yield), FE% (Fermentation Efficiency), EP (Ethanol productivity) and YoE (Yield of Ethanol recovered from Kg of dry substrate).

### 3.3 Molecular identification

**Fig. 4:**
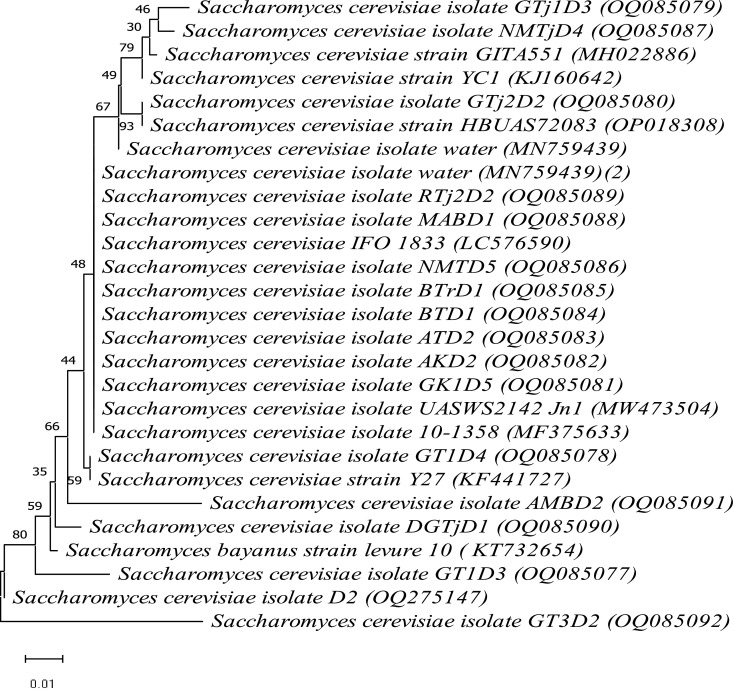
Phylogenetic tree of investigated ethanologenic *S. cerevisiae* strains (accession numbers from OQ085077-OQ085092) and selected closely related strains scored ≥ 99 % identity similarity retrieved from NCBI database.

Selected stress-tolerant and bioethanol producing isolates were identified using the ITS regions of the rRNA gene as a barcode. Species level was assigned based on BLASTn identity score match ≥ 99 % against sequences in the GenBank. Ethanologenic yeast were identified as *S. cerevisiae* isolates. Then, the sequence of each isolate was deposited in GenBank in the NCBI database, and accession numbers were obtained (Fig. 4). So as to determine their phylogenetic position and infer their evolutionary history, sequences of the current isolates and related strains were retrieved from GenBank, aligned with ClustalW, and a phylogenetic tree was constructed using MEGA software version 11.0 [109] (Fig. 4). The phylogenetic tree was constructed from the evolutionary distance data calculated from the Jukes-Cantor model [110] using the neighbor-joining method [111].

## 4 Conclusion

The results of the present study demonstrate that traditional fermented beverages are an important source of ethanologenic yeasts. Molecular identification revealed that all selected isolates belong to the species *S. cerevisiae*. The screening of these *S. cerevisiae* strains for ideal properties of ethanologenic yeast, such as the ability to ferment and assimilate carbohydrate molecules, grow and survive under different stress conditions, and produce ethanol from glucose evidenced that these strains are applicable in bioethanol industries. Ethanol production evaluation using banana peel as a substrate showed that all tested isolates produced a considerable amount of ethanol, ranging from 9.1 ± 0.6 to 12.9 ± 1.3 g/L and sugar to ethanol conversion efficiency varied between 54.8 ± 3.9 to 77.6 ± 6.0 %. This ensures that they are a good candidate for ethanol production using lignocellulosic biomass as a substrate. Study on optimization of the fermentation process is recommended.

## Authors contribution

Conception of the research idea, design of the study, acquisition, analysis and interpretation of data and manuscript drafting were done by DB. B.A. and A.T. have supervised the laboratory work, edit and approve the manuscript to be submitted.

## Funding source

This research did not receive any specific grant from funding agencies in the public, commercial, or not-for-profit sectors.

## Declaration of Competing Interest

The authors declare that they have no known competing financial interests or personal relationships that could have appeared to influence the work reported in this paper.

## Data Availability

Data will be made available on request.

## Appendix Supplementary materials

Image, application 1
