# Supplementary material for: Isolation, screening and identification of ethanol producing yeasts from Ethiopian fermented beverages
Source: Biotechnol Rep (Amst). 2023 Oct 4;40:e00815. doi: 10.1016/j.btre.2023.e00815 (PMC10590766; doi:10.1016/j.btre.2023.e00815)
Supplement: Supplementary file 1 [file mmc1.docx]

**Annex - Supplementary data**


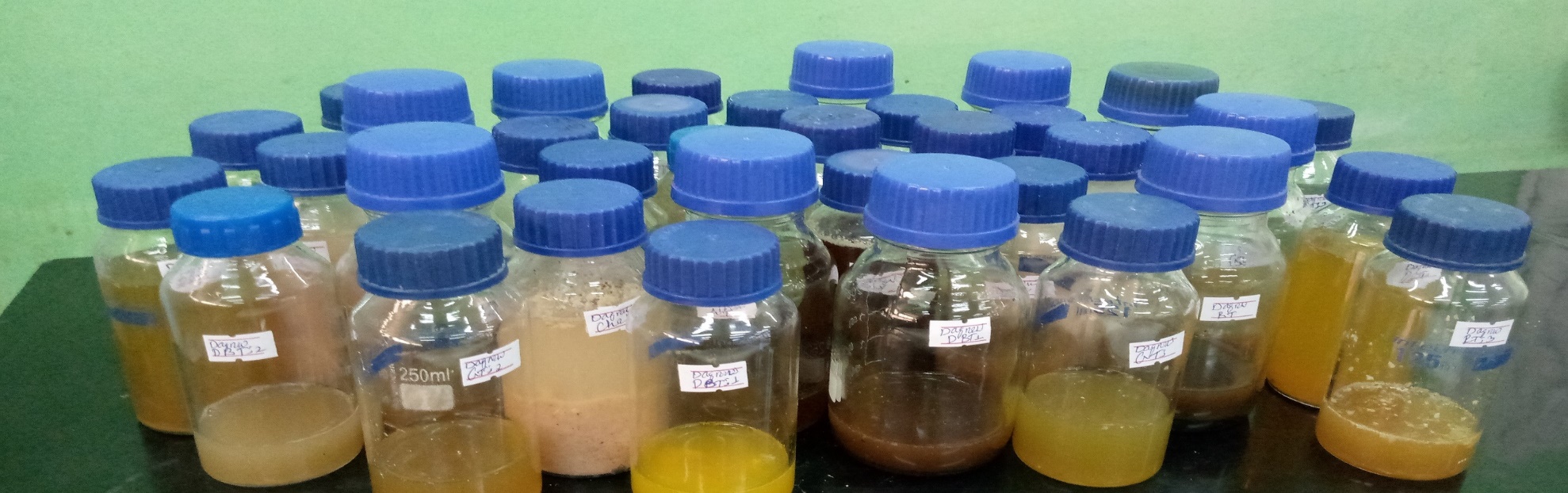


**Fig. 1** Sample of traditional fermented beverages collected


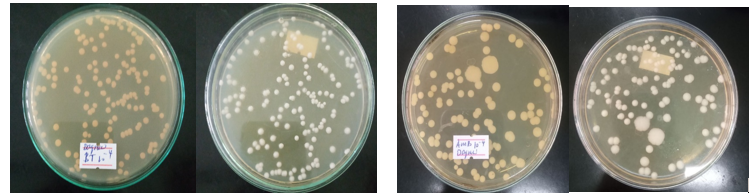


**Fig. 2** Typical colony picture for yeast isolates

**Table. 1.** Carbohydrate fermentation performance of isolate after 48h of incubation at 30 ^O^C

| **Isolate** | **Types of Carbohydrate** | | | | | **ANOVA** | |
| --- | --- | --- | --- | --- | --- | --- | --- |
|  | **Glucose** | **Sucrose** | **Maltose** | **Galactose** | **Lactose** | **F value** | **P value** |
| GT1D1 | 5±0.0^b^ | 4.3±0.6^b^ | 3.3±0.3^a^ | 2.7±0.6^a^ | - | 17.18 | 0.00 |
| GT1D2 | 5±0.0^b^ | 3.3±1.4^a^ | 3.5±0.9^a^ | 1.8±0.8^b^ | - | 5.88 | 0.02 |
| GT1D3 | 5±0.0^c^ | 5±0.0^c^ | 2.8±0.3^b^ | 1.3±0.3^a^ | - | 231.17 | 0.00 |
| GT1D4 | 4.7±0.6^b^ | 4.7±0.6^b^ | 2.8±0.8^a^ | 2.8±0.8^a^ | - | 7.33 | 0.01 |
| GT3D2 | 5±0.0^c^ | 4.7±0.6^b^ | 3.5±1.3^b^ | 1±0.9^a^ | - | 13.91 | 0.00 |
| GTj1D2 | 5±0.0^b^ | 4.2±0.8^b^ | 4.5±0.5^b^ | 0.70±0.3^a^ | - | 51.03 | 0.00 |
| GTj1D3 | 5±0.0^b^ | 4.2±0.3^b^ | 3.7±0.3^a^ | 3.2±0.8^a^ | - | 9.78 | 0.05 |
| GTj2D2 | 5±0.0^b^ | 4.8±0.3^b^ | 2±0.5^a^ | 2±0.9^a^ | - | 31.46 | 0.00 |
| GTj2D3 | 5±0.0^c^ | 4.2±0.3^b^ | 3.7±0.3^b^ | 1.8±0.3^a^ | - | 86.22 | 0.00 |
| GTj3D1 | 5±0.0^a^ | 3.8±1.04^a^ | 4.2±0.3^a^ | - | - | 50.76 | 0.00 |
| GTj3D2 | 4.8±0.3^b^ | 4.2±0.8^b^ | 4.3±1.2^b^ | 0.3±0.6^a^ | - | 22.14 | 0.00 |
| GTj3D3 | 4.7 ±0.6^b^ | 4.5±0.5^b^ | 4.7±0.6^b^ | 0.7±0.3^a^ | - | 46.75 | 0.00 |
| GTj3D5 | 5±0.0^b^ | 3.8±2.0^b^ | 3.7±2.3^b^ | 0.5±0.0^a^ | - | 4.72 | 0.03 |
| GB2D3 | 4.5±0.5^c^ | 2.2±1.0^b^ | 1.5±0.5^b^ | 0.3±0.3^a^ | - | 22.18 | 0.00 |
| GB2D5 | 5±0.0^b^ | 3.8±0.3^b^ | 4.2±0.8^b^ | 1±1.0^a^ | - | 21.73 | 0.00 |
| AKD2 | 5±0.0^b^ | 3.2±0.8^b^ | 1.8±1.3^a^ | 0.5±0.5^a^ | - | 18.33 | 0.00 |
| ATD1 | 5±0.0^d^ | 3.5±0.5^c^ | 2±0.9^b^ | 0.6±0.6^a^ | - | 32.14 | 0.00 |
| ATD2 | 5±0.0^b^ | 4.5±0.5^b^ | 3.8±0.8^b^ | 0.50±0.5^a^ | - | 45.62 | 0.00 |
| GK1D5 | 4.8±0.3^c^ | 4±0.9^c^ | 3±1.0^b^ | 0.5±0.0^a^ | - | 23.09 | 0.00 |
| BTD1 | 5±0.0^c^ | 3.3±0.3^b^ | 3.2±0.8^b^ | 0.5±0.5^a^ | - | 45.33 | 0.00 |
| BTD4 | 4.6±0.8^c^ | 2.3±2.4^b^ | 3±1.8^b^ | 1±0.9^a^ | - | 2.6 | 0.12 |
| BTjD1 | 5±0.0^c^ | 4.7±0.6^c^ | 2±1.3^b^ | 1±0.0^a^ | - | 22.40 | 0.00 |
| BTjD2 | 5±0.0^b^ | 4.2±0.3^b^ | 4.7±0.3^b^ | 1.8±1.9^a^ | - | 6.55 | 0.00 |
| BTrD1 | 5±0.0^b^ | 3.9±0.4^b^ | 3.7±1.2^b^ | 1.5±0.9^a^ | - | 11.63 | 0.00 |
| BTrD3 | 5 ±0.0^c^ | 3.3±1.0^c^ | 2.3±1.0^b^ | 0.8±0.3^a^ | - | 16.33 | 0.00 |
| NMTD2 | 4.8±0.3^c^ | 4.2±0.3^c^ | 2.2±0.8^b^ | 0.2±0.3^a^ | - | 66.46 | 0.00 |
| NMTD5 | 5±0.0^b^ | 4.2 ±0.8^b^ | 3.7±2.3^b^ | 1.2±0.8^a^ | - | 5.03 | 0.03 |
| NMTjD3 | 5±0.0^b^ | 4.8±0.3^b^ | 4.5±0.9^b^ | 0.3±0.6^a^ | - | 51.24 | 0.00 |
| NMTjD4 | 5±0.0^b^ | 4.5±0.5^b^ | 2±1.5^a^ | 1±0.9^a^ | - | 13.77 | 0.00 |
| NMKD2 | 5±0.0^c^ | 3.8±0.3^c^ | 1.5±0.9^b^ | 0.3±0.3^a^ | - | 59.39 | 0.00 |
| MABD1 | 5±0.0^c^ | 3.3±0.3^b^ | 1.2±0.8^a^ | 0.3±0.6^a^ | - | 53.64 | 0.00 |
| MABD3 | 4.9±0.2^c^ | 3.3±0.6^b^ | 1.5±0.9^a^ | - | - | 49.17 | 0.00 |
| RTj1D5 | 5 ±0.0^b^ | 2.2±0.3^a^ | 1.2±0.8^a^ | 0.7±1.2^a^ | - | 22.44 | 0.00 |
| RTj2D2 | 4.5±0.9^c^ | 3±1.32^b^ | 2.3±1.15^b^ | 0.3±0.6^a^ | - | 8.61 | 0.01 |
| RTj2D4 | 4.5±0.4^b^ | 3.5±0.5^b^ | 2.5±0.5^a^ | 1.3±0.6^a^ | - | 21.58 | 0.00 |
| DBTj1D1 | 3.7±0.3^b^ | 3±1.0^b^ | 0.3±0.3^a^ | 0.8±0.3^a^ | - | 25.31 | 0.00 |
| DGTD2 | 5±0.0^b^ | 5±0.0^b^ | 3.2±1.4^b^ | 1.5±0.5^a^ | - | 14.57 | 0.00 |
| DGTjD1 | 5±0.0^b^ | 5±0.0^b^ | 4.3±1.2^b^ | 2.7±0.8^a^ | - | 7.59 | 0.01 |
| AMBD2 | 4.7±0.5^b^ | 5±0.0^b^ | 4±0.0^b^ | 1.5±0.5^a^ | - | 65.96 | 0.00 |

**Key:** Mean values superscripted with the same letter across the raw are not significantly
 different at p≤ 0.05.

(-) denotes negative for fermentation


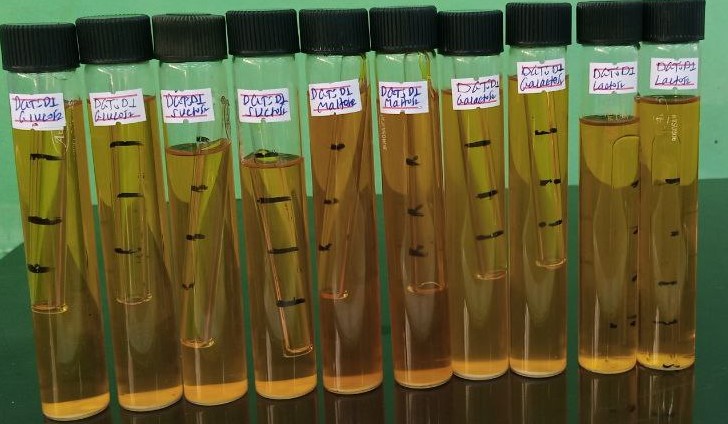


**Fig. 3** Typical picture for carbohydrate fermentation of isolates

**Table 9.** Carbohydrate assimilation profile of pre-selected isolates

| Isolate | **Carbohydrate Molecules** | | | | | | | | |
| --- | --- | --- | --- | --- | --- | --- | --- | --- | --- |
|  | Glucose | Sucrose | Maltose | Galactose | Lactose | Mannose | Starch | Mannitol | Arabinose |
| GT1D1 | + | + | + | + | - | + | - | - | - |
| GT1D2 | + | + | + | + | - | + | - | - | - |
| GT1D3 | + | + | + | + | - | + | - | - | - |
| GT1D4 | + | + | + | + | - | + | - | - | - |
| GT3D2 | + | + | + | + | - | + | - | - | - |
| GTj1D2 | + | + | + | + | - | + | - | - | - |
| GTj1D3 | + | + | + | + | - | + | - | - | - |
| GTj2D1 | + | + | + | + | - | + | - | - | - |
| GTj2D2 | + | + | + | + | - | + | - | - | - |
| GTj2D3 | + | + | + | + | - | + | - | - | - |
| GTj3D3 | + | + | + | + | - | + | - | - | - |
| GTj3D5 | + | + | + | + | **-** | **+** | - | - | - |
| GB2D3 | + | + | + | + | **-** | **+** | - | - | - |
| GB2D5 | + | + | + | + | **-** | **+** | - | - | - |
| ATD1 | + | + | + | + | **-** | **+** | - | - | - |
| ATD2 | + | + | + | + | **-** | + | - | - | - |
| AKD2 | + | + | + | + | **-** | + | - | - | - |
| GKID5 | + | + | + | + | **-** | + | - | - | - |
| NMTD2 | + | + | + | + | **-** | + | - | - | - |
| NMTD5 | + | + | + | + | **-** | + | - | - | - |
| BTrD1 | + | + | + | + | **-** | + | - | - | - |
| BTrD3 | + | + | + | + | **-** | + | **-** | **-** | **-** |
| BTjD1 | + | + | + | + | **-** | + | **-** | **-** | **-** |
| BTjD2 | + | + | + | + | **-** | + | **-** | **-** | **-** |
| NMTjD3 | + | + | + | + | **-** | + | **-** | **-** | **-** |
| NMTjD4 | + | + | + | + | **-** | + | **-** | **-** | **-** |
| NMKD2 | + | + | + | + | **-** | + | **-** | **-** | **-** |
| RTj2D2 | + | + | + | + | **-** | + | **-** | **-** | **-** |
| RTj2D4 | + | + | + | + | **-** | + | **-** | **-** | **-** |
| MABD1 | + | + | + | + | **-** | + | **-** | **-** | **-** |
| MABD3 | + | + | + | + | **-** | + | **-** | **-** | **-** |
| DBTjD1 | + | + | + | + | **-** | + | **-** | **-** | **-** |
| DGTjD1 | + | + | + | + | **-** | + | **-** | **-** | **-** |
| DGTD2 | + | + | + | + | **-** | + | **-** | **-** | **-** |
| AMBD2 | + | + | + | + | **-** | + | **-** | **-** | **-** |

**Key:** (+) denotes assimilation positive whereas (-) is negative


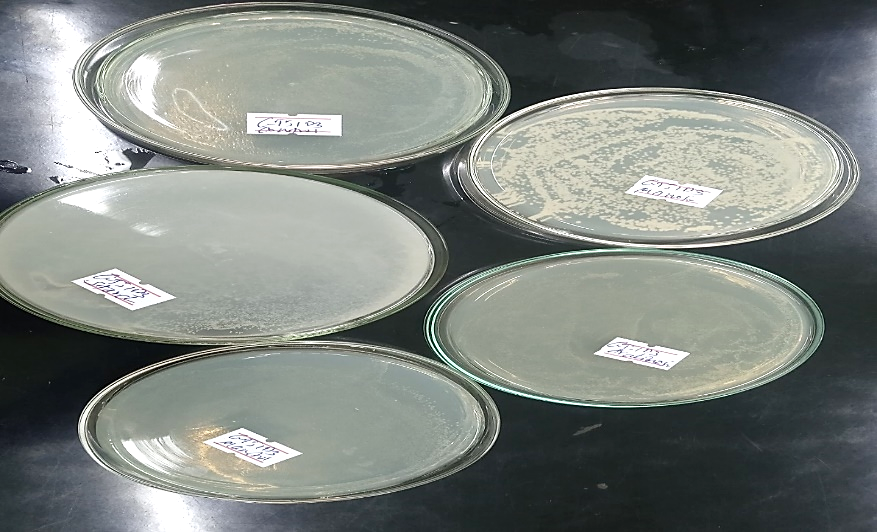


**Fig. 4** Typical picture for assimilation of carbohydrate molecules

**Table 3**. Glucose fermentation performance of isolates at different pH values after 48hrs of incubation at 30 ^O^C.

| **Isolate** | pH values | | | | | | | | ANOVA | |
| --- | --- | --- | --- | --- | --- | --- | --- | --- | --- | --- |
|  | **3** | **3.5** | **4** | **4.5** | **5** | **5.5** | **6** | **6.5** | **F-value** | **P-value** |
| GT1D1 | 4.5±0.50^a^ | 5±0.00^a^ | 5±0.00^a^ | 5±0.00^a^ | 4.5±0.50^a^ | 0.47±0.58^b^ | 0.67±0.29^b^ | 0±0.00^b^ | 217.42 | 0.00 |
| GT1D2 | 3.7±1.44^a^ | 4.7±0.58^a^ | 5±0.00^a^ | 5±0.00^a^ | 3.9±0.96^a^ | 3.3±1.53^a^ | 2.3±0.58^b^ | 0.7±0.29^b^ | 8.68 | 0.00 |
| GT1D3 | 3±0.00^a^ | 3.6±0.85^a^ | 4±1.00^a^ | 4.7±0.58^a^ | 3.8±1.61^a^ | 0.9±0.36^b^ | 0.4±0.36^b^ | 0.17±0.29^b^ | 15.50 | 0.00 |
| GT1D4 | 3.2±0.29^a^ | 3.7±0.58^b^ | 4.7±0.58^b^ | 4.8±0.29^b^ | 3.7±0.58^b^ | 1.3±0.58^c^ | 0.93±0.12^d^ | 0±0.00^d^ | 51.49 | 0.00 |
| GT3D2 | 3.4±1.65^a^ | 4±1.00^a^ | 4±0.50^a^ | 4.2±0.58^a^ | 3.7±1.15^a^ | 2±0.50^a^ | 2.2±0.29^a^ | 1.3±0.58^b^ | 4.55 | 0.00 |
| GTj1D2 | 4.2±0.76^a^ | 4.8±0.29^a^ | 5±0.00^a^ | 5±0.00^a^ | 5±0.00^a^ | 4.8±0.29^a^ | 1±1.32^b^ | 1±1.00^b^ | 21.79 | 0.00 |
| GTj1D3 | 5 ±0.00^a^ | 5±0.00^a^ | 5±0.00^a^ | 5±0.00^a^ | 5±0.00^a^ | 3.5±0.50^a^ | 1.3±1.53^b^ | 0.17±0.29^b^ | 34.14 | 0.00 |
| GTj2D2 | 4.5±0.87^a^ | 5±0.00^a^ | 5±0.00^a^ | 5±0.00^a^ | 4.8±0.29^a^ | 3.6±0.98^a^ | 3±0.500^b^ | 2.3±0.29^b^ | 12.43 | 0.00 |
| GTj2D3 | 4.2±0.58^a^ | 5±0.00b | 5±0.00b | 5±0.00b | 5±0.00b | 4.5±0.00b | 3.5±0.50a | 2.2±0.29c | 37.07 | 0.00 |
| GTj3D1 | 4.8±0.29^a^ | 5±0.00a | 4.8±0.29a | 5±0.00a | 3.3±0.58a | 1.7±0.58b | 1.3±0.29b | 0.83±0.29c | 79.13 | 0.00 |
| GTj3D2 | 3.7±1.04^a^ | 4.7±0.58a | 4.7±0.58a | 4.7±0.58a | 5±0.00a | 3.8±1.26a | 2.7±1.15b | 1±0.50b | 8.38 | 0.00 |
| GTj3D3 | 3.8±0.29a | 4.8±0.29b | 4.8±029b | 5±0.00b | 5±0.00b | 4.2±0.29b | 4.2±0.58b | 2.2±0.76c | 17.37 | 0.00 |
| GTj3D5 | 4±0.50a | 4.8±0.29a | 5±0.00a | 5±0.00a | 4.8±0.29a | 3.3±0.58b | 2.7±0.76b | 2.2±0.76b | 16.04 | 0.00 |
| GB2D3 | 3.7±0.29a | 4±1.00a | 4.2±0.76a | 4.8±0.29a | 3.2±1.75a | 2±0.00b | 2±1.00b | 1.2±0.76b | 6.11 | 0.00 |
| GB2D5 | 2.3±1.53a | 3±1.80a | 3.3±1.75a | 4.7±0.58a | 3.7±1.52a | 3.2±1.61a | 1.2±0.76a | 1±0.50a | 2.52 | 0.06 |
| AKD2 | 5±0.00^a^ | 5±0.00^a^ | 5±0.00a | 5±0.00a | 4.5±0.50a | 3.8±0.35b | 3.3±0.58b | 2.1±0.40c | 32.09 | 0.00 |
| ATD1 | 5±0.00^a^ | 5±0.00^a^ | 5±0.00a | 5±0.00a | 4.8±0.29a | 4.5±0.50a | 3.5±0.50b | 1.9±0.17c | 48.47 | 0.00 |
| ATD2 | 4.7±0.58^a^ | 5±0.00^a^ | 5±0.00a | 5±0.00a | 5±0.00a | 4.8±0.29a | 4±1.00a | 2.2±2.25b | 3.60 | 0.02 |
| GK1D5 | 4.3±0.29a | 4.7±0.58^a^ | 5±0.00a | 5±0.00a | 5±0.00a | 4.7±0.58a | 3.2±0.29b | 2.5±0.50b | 20.14 | 0.00 |
| BTD1 | 4.8±0.29^a^ | 5±0.00^a^ | 5±0.00a | 5±0.00a | 4.5±0.87a | 4±1.00a | 2.2±0.76b | 1.2±0.25b | 20.67 | 0.00 |
| BTD4 | 4.6±0.75^a^ | 4.7±0.58a | 4.6±0.75a | 5±0.00a | 3.9±1.01^a^ | 3.6±0.40a | 3.0±1.27a | 1.8±0.76b | 5.54 | 0.00 |
| BTjD1 | 5±0.00a | 5±0.00a | 5±0.00a | 5±0.00a | 4.7±0.58a | 4.2±0.58a | 3.3±0.29b | 2.3±0.29c | 28.44 | 0.00 |
| BTjD2 | 4.8±0.29a | 5±0.00a | 5±0.00a | 5±0.00a | 5±0.00a | 5±0.00a | 3.8±0.29b | 2.7±0.76c | 23.56 | 0.00 |
| BTrD1 | 5±0.00a | 5±0.00a | 5±0.00a | 5±0.00a | 5±0.00a | 4.2±0.58a | 3.6±0.51b | 2±1.00c | 17.78 | 0.00 |
| BTrD3 | 5±0.00a | 5±0.00a | 5±0.00a | 5±0.00a | 5±0.00a | 4±1.00a | 2.5±1.36b | 1.5±1.00c | 9.01 | 0.00 |
| NMTD2 | 4.5±0.81a | 5±0.00a | 5±0.00a | 5±0.00a | 4.3±0.58a | 3.6±0.53a | 2.8±1.04b | 1.3±0.57b | 15.35 | 0.00 |
| NMTD5 | 5±0.00a | 5±0.00a | 5±0.00a | 5±0.00a | 5±0.00a | 5±0.00a | 3.7±0.58b | 1±0.50c | 83.59 | 0.00 |
| NMTjD3 | 4.7±0.58a | 5±0.00a | 5±0.00a | 5±0.00a | 5±0.00a | 4±0.50a | 3.1±0.81b | 2.3±0.58b | 16.17 | 0.00 |
| NMTjD4 | 4.3±0.58a | 4.7±0.58a | 5±0.00a | 5±0.00a | 5±0.00a | 3.7±0.26a | 0.3±0.58b | 0±0.00b | 99.73 | 0.00 |
| NMKD2 | 5 ±0.00a | 5±0.00a | 5±0.00a | 5±0.00a | 4.3±0.64a | 3±0.50b | 2.6±0.93b | 1.2±0.76c | 24.13 | 0.00 |
| MABD1 | 4.2±0.29a | 5±0.00b | 5±0.00b | 5±0.00b | 5±0.00b | 5±0.00b | 4.7±0.58b | 4±0.00a | 10.03 | 0.00 |
| MABD3 | 3.2±0.29a | 3.7±0.29a | 4.6±0.69b | 5±0.00b | 5±0.00b | 5±0.00b | 4.3±0.58 | 3.9±0.17a | 15.34 | 0.00 |
| RTj1D5 | 4.8±0.29a | 4.3±0.58a | 4±1.00a | 4.4±0.98a | 4.3±1.15a | 3.4±0.65a | 2.2±0.29b | 1.8±0.29b | 6.87 | 0.00 |
| RTj2D2 | 5 ±0.00a | 5 ±0.00a | 4.5 ±0.86a | 5 ±0.00a | 5 ±0.00a | 5 ±0.00a | 4 ±1.00a | 3.2 ±1.08b | 3.57 | 0.00 |
| RTj2D4 | 4.8±0.29a | 4.7±0.58a | 4.7±0.58a | 4±1.73a | 3.8±2.02a | 3.3±1.26a | 3.3±1.15a | 2.3±0.58a | 1.59 | 0.20 |
| RTj3D3 | 5±0.00a | 5±0.00a | 5±0.00a | 5±0.00a | 5±0.00a | 4.8±0.25a | 3.5±0.86b | 2.8±0.76b | 12.51 | 0.00 |
| DBTj1D1 | 2.5±1.81a | 3.3±0.58b | 4.2±0.29b | 4.7±0.58b | 3.7±1.53b | 2.3±1.53a | 0.7±0.76a | 0.17±0.29a | 6.64 | 0.00 |
| DMTD2 | 3.8±0.61a | 5±0.00a | 5±0.00a | 5±0.00 | 5±0.00 | 4.2±0.75a | 3.2±0.29b | 2.4±0.69b | 16.10 | 0.00 |
| DGTD2 | 5±0.00a | 5±0.00a | 5±0.00a | 5±0.00a | 4.8±0.29a | 3.9±0.36b | 3.8±0.29b | 1.3±0.58c | 61.70 | 0.00 |
| DGTjD1 | 5±0.00^a^ | 5±0.00^a^ | 4.8±0.29^a^ | 5±0.00^a^ | 4.4±1.04^a^ | 3.5±0.15^b^ | 3.1±0.17^b^ | 2.4±0.85^b^ | 13.00 | 0.00 |
| AMBD2 | 5±0.00^a^ | 5±0.00^a^ | 5±0.00^a^ | 5±0.00^a^ | 5±0.00^a^ | 3.8±0.29^b^ | 3±0.55^b^ | 2.3±0.76^c^ | 29.35 | 0.00 |

**Key:** Mean values superscripted with different letter across the raw are significantly different

**Table 4.** Dry-weight of pre-selected isolate at different pH values after 72 hrs of incubation at 30 ^O^C.

| **Isolate** | pH values | | | | | | | | ANOVA | |
| --- | --- | --- | --- | --- | --- | --- | --- | --- | --- | --- |
|  | **3** | **3.5** | **4** | **4.5** | **5** | **5.5** | **6** | **6.5** | **F-value** | **P-value** |
| GT1D1 | 56.3±2.08a | 62.3±2.52a | 74±2.00b | 82.3±2.52b | 76.3±3.21b | 64.3±2.52a | 61.3±6.65a | 54±2.65c | 27.42 | 0.00 |
| GT1D2 | 62.7±2.08a | 64.7±4.16a | 72±4.58a | 79.3±1.15b | 82.3±2.52b | 80.3±5.68b | 73.3±3.05b | 72.3±2.52a | 12.47 | 0.00 |
| GT1D3 | 67.3±2.52^a^ | 72.3±2.52^a^ | 84±1.73^b^ | 85.7±1.52^b^ | 88±4.00^b^ | 85.3±4.2^b^ | 68.7±5.68^a^ | 54.3±1.15^c^ | 40.32 | 0.00 |
| GT1D4 | 74.3±3.78a | 76±6.93a | 81.7±3.51a | 91.3±4.04b | 93.3±2.88b | 80.3±2.52a | 79.7±5.50a | 70.3±5.50a | 9.17 | 0.00 |
| GT3D2 | 62.3±2.52a | 64.3±3.05a | 74.7±1.53b | 77.3±3.05b | 73±2.65b | 74.7±2.08b | 67.7±4.04a | 66±5.29a | 9.03 | 0.00 |
| GTj1D2 | 78.7±3.05a | 82.3±7.09a | 84±5.29a | 87.7±4.50a | 86.7±2.89a | 73±1.00b | 69.3±1.15b | 65.3±4.16b | 12.18 | 0.00 |
| GTj1D3 | 61.3±4.04a | 62.7±3.05a | 65.7±4.04b | 75.7±1.15b | 74±3.46b | 73.7±5.51b | 71.7±2.89b | 65±2.64b | 7.59 | 0.00 |
| GTj2D2 | 79.7±1.53a | 85.7±1.15a | 91±4.58b | 93.3±2.89b | 86.3±2.08b | 80.7±1.15a | 78±4.36a | 75.7±3.58c | 13.30 | 0.00 |
| GTj2D3 | 76.7±3.05a | 78±4.36a | 81.3±3.21a | 82.7±4.04a | 78±2.00a | 75±1.73a | 71.7±3.51b | 67.7±2.51b | 7.20 | 0.00 |
| GTj3D1 | 58.7±4.16a | 60±6.25a | 69.7±1.53b | 75.3±3.05b | 80±5.00b | 79±7.21b | 71.7±3.78b | 69.7±1.53b | 9.31 | 0.00 |
| GTj3D2 | 70.3±3.51a | 75.3±5.86b | 81±5.57b | 84±5.57b | 82.7±2.51b | 77.7±2.51b | 65±2.64a | 58.7±3.21a | 14.04 | 0.00 |
| GTj3D3 | 65±8.89a | 79.7±3.51b | 82.7±2.52b | 85.3±4.16c | 83.3±5.86b | 71.3±1.53b | 60.3±1.53a | 52.3±6.43a | 18.41 | 0.00 |
| GTj3D5 | 78.3±2.89a | 79±2.00 | 85±2.00b | 88.7±4.16b | 86±4.00b | 78±2.65a | 74.7±3.05a | 70±3.00c | 12.45 | 0.00 |
| GB2D3 | 76.7±4.04a | 80±5.57b | 83.7±4.04b | 90±3.00b | 88±4.35b | 79±1.73b | 72.3±4.73a | 66.7±3.78a | 11.08 | 0.00 |
| GB2D5 | 50.7±7.57a | 61.7±7.57a | 71.7±2.89b | 75±2.65b | 73.7±1.53b | 71±1.73 | 68.3±1.53b | 65±2.00b | 10.82 | 0.00 |
| AKD2 | 77±2.65a | 80.3±1.53a | 85.7±1.15b | 91.3±4.04b | 84.7±0.58b | 79.7±5.50b | 72±2.00c | 66.3±3.05c | 21.31 | 0.00 |
| ATD1 | 76.7±3.05b | 83.7±5.13b | 89±1.73a | 90±0.00a | 84.3±4.04a | 77±2.65b | 69.7±0.58c | 68.3±2.89c | 23.09 | 0.00 |
| ATD2 | 63±3.00a | 68.7±1.53b | 74.3±1.15b | 78.3±5.85b | 78±2.00b | 73±4.58b | 68.3±5.86b | 53.7±8.50c | 9.22 | 0.00 |
| GK1D5 | 75.3±3.05a | 79.3±5.50a | 84.7±1.53a | 86.7±1.53a | 77.3±3.05a | 70±5.57a | 65.7±8.14b | 58.7±9.86b | 8.73 | 0.00 |
| BTD1 | 82.7±3.05a | 83±4.36a | 87±3.00a | 86.3±3.51a | 85±2.00a | 79.3±1.15a | 72±3.46b | 67±3.00b | 16.37 | 0.00 |
| BTD4 | 68±5.29a | 74±1.00a | 85.7±5.13b | 82.3±2.52b | 80.3±5.86b | 73±3.00a | 73.7±3.21a | 62.7±4.62a | 10.13 | 0.00 |
| BTjD1 | 72.7±4.04b | 76.3±0.58a | 83±4.36a | 85.3±5.03a | 81±4.58a | 73±2.65b | 69.7±4.72b | 58.7±4.16c | 13.68 | 0.00 |
| BTjD2 | 76.7±5.77a | 78.3±4.72a | 81.3±4.04a | 95.7±5.13b | 86.3±3.51b | 82±2.00a | 76±4.00a | 73±3.00c | 8.82 | 0.00 |
| BTrD1 | 72.3±3.21* | 76±3.60 | 80.7±3.51 | 84±3.60* | 82.3±5.50 | 80±4.58 | 78.7±2.30 | 74±3.60 | 3.37 | 0.02 |
| BTrD3 | 65.3±3.21a | 73.3±4.16c | 78±2.00b | 80.7±1.15b | 84±2.00b | 76±2.00c | 73±3.00c | 71.7±2.89a | 13.75 | 0.00 |
| NMTD2 | 78±2.00a | 81.7±3.51a | 88±2.00b | 90.7±4.04b | 89±3.60b | 81±3.60a | 77±1.73a | 75.7±4.04a | 10.00 | 0.00 |
| NMTD5 | 68.7±1.15a | 77±2.00b | 87.3±2.31c | 87.3±4.93c | 92±4.00c | 82.7±2.52b | 76.7±1.53b | 70.7±1.15a | 29.53 | 0.00 |
| NMTjD3 | 69±1.73a | 74±1.73b | 89±8.18c | 90±6.08c | 83±3.00c | 76.3±2.08b | 76.7±2.89b | 70±2.65a | 11.21 | 0.00 |
| NMTjD4 | 71.3±4.16 | 77.3±2.51 | 77±3.60 | 87.3±2.51 | 84.3±2.08 | 77±3.00 | 68.7±2.31 | 66±2.65 | 18.83 | 0.00 |
| NMKD2 | 64.3±3.78a | 72±3.46b | 75±1.00b | 86±5.29c | 87.7±4.04d | 78.3±2.08c | 77.7±2.52c | 75±1.73b | 15.69 | 0.00 |
| MABD1 | 47±3.00a | 49±3.46b | 60±6.93b | 66.3±3.51b | 62±9.54b | 60±0.00b | 57±2.52b | 54.7±6.11b | 4.76 | 0.05 |
| MABD3 | 60±3.00a | 69.3±5.13c | 77±1.00b | 80.3±4.50b | 74.3±2.52b | 71±1.73c | 69.3±2.08c | 68.3±2.89c | 11.66 | 0.00 |
| RTj1D5 | 80±4.36 | 83.3±6.43 | 89.7±6.43 | 92.8±4.93 | 88.7±2.30 | 79.3±6.65 | 82.7±5.86 | 80.3±0.58 | 2.93 | 0.04 |
| RTj2D2 | 54.7 ±3.05a | 72.7±6.80c | 77.3±2.52c | 84±2.00c | 84.3±6.43c | 80.3±5.13c | 74.7±4.50c | 70.3±4.50b | 12.64 | 0.00 |
| RTj2D4 | 78.7±5.50a | 85.3±4.62b | 88.7±8.08b | 91±9.53b | 79±1.73b | 74.3±5.68a | 70.3±4.50a | 65±4.36a | 6.92 | 0.00 |
| RTj3D3 | 82.3±5.50b | 83.7±4.04b | 87.3±2.30c | 93±5.29c | 94±2.00c | 78±2.00a | 77.7±2.52a | 69.7±5.68a | 12.76 | 0.00 |
| DBTj1D1 | 46.7±3.51a | 58.3±1.53b | 62.3±2.52b | 64.7±4.04b | 68.3±2.89c | 71±4.00c | 57.7±3.21b | 45.7±3.78a | 24.05 | 0.00 |
| DMTD2 | 51.7±3.78a | 58.3±3.51a | 65.3±4.50b | 72.3±2.52b | 57.7±4.50a | 57.7±4.04a | 57±6.00a | 55.3±5.03a | 6.61 | 0.00 |
| DGTD2 | 67.7±2.52a | 71±3.60a | 76.7±7.64b | 81.7±2.89b | 79.7±5.50b | 78.3±5.68b | 68±3.46a | 65±5.00a | 5.19 | 0.00 |
| DGTjD1 | 65.3±1.53a | 73.3±4.16a | 77±3.00a | 79.7±2.52b | 74.7±2.52a | 74.7±5.03a | 71±5.57a | 68±3.46a | 4.81 | 0.00 |
| AMBD2 | 66.3±3.21^a^ | 69.7±2.52^a^ | 72.3±2.08^a^ | 79.7±5.03^b^ | 72.7±3.21^a^ | 66±5.29^a^ | 53.3±4.72^c^ | 48.7±4.16^c^ | 20.56 | 0.00 |

**Key:** Mean values superscripted with different letter across the raw are significantly different


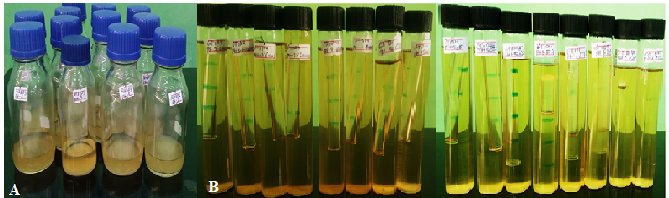


**(B)**

**(A)**

**Fig.** **5** Typical picture for growth (A) and fermentation performance of isolate at different pH values (B)

**Table 5.** Fermentation performance of isolate in terms of replaced liquid in 5ml Durham tube grown at different temperature after 48 hrs of incubation.

| Isolate | Incubation Temperature in ^0^C | | | | | ANOVA | |
| --- | --- | --- | --- | --- | --- | --- | --- |
|  | 20 | 25 | 30 | 35 | 40 | F value | P value |
| GT1D1 | 3.5±0.50^a^ | 4.3 ±0.58^b^ | 4.7±0.58^b^ | 5±0.00^b^ | - | 5.42 | 0.03 |
| GT1D2 | 2.8±1.44^a^ | 4.2±0.68^b^ | 5±0.00^b^ | 5±0.00^b^ | - | 4.92 | 0.03 |
| GT1D3 | 2.2±1.75^a^ | 5±0.00^b^ | 5±0.00^b^ | 5±0.00^b^ | - | 7.81 | 0.01 |
| GT1D4 | 2.5±0.46^a^ | 4.7±0.29^b^ | 4.7±0.58^b^ | 5±0.00^b^ | - | 25.91 | 0.00 |
| GT3D2 | 1.1 ±0.12^a^ | 4.1±0.51^b^ | 5±0.00^b^ | 5±0.00^b^ | - | 150.67 | 0.00 |
| GTj1D2 | 1.5±0.50^a^ | 3.5±0.87^b^ | 5±0.00^c^ | 3.8±0.29^b^ | - | 23.46 | 0.00 |
| GTj1D3 | 3±0.50^a^ | 4±0.00^b^ | 5±0.00^b^ | 2.6±0.53^a^ | - | 28.19 | 0.00 |
| GTj2D2 | 0.8±0.61^a^ | 2.9±0.85^b^ | 5±0.00^c^ | 2.06±0.93^b^ | - | 19.03 | 0.00 |
| GTj2D3 | 0.3±0.58^a^ | 2.7±1.04^b^ | 5±0.00^b^ | 3.4±1.97^b^ | - | 8.53 | 0.00 |
| GTj3D1 | 2.3±0.29^a^ | 4.1±0.12^b^ | 5±0.00^c^ | 4±0.50^b^ | - | 42.60 | 0.00 |
| GTj3D2 | 0.5 ±0.87a | 1.8±0.29^b^ | 4.8±0.29c | 0.2±0.29^a^ | - | 54.22 | 0.00 |
| GTj3D3 | 1.7±1.15a | 3.5±0.50b | 4.7±0.58b | 1±1.00a | - | 11.61 | 0.00 |
| GTj3D5 | 0.8±0.76^a^ | 3.8±1.04^b^ | 5±0.00b | 3.7±1.25b | - | 11.69 | 0.00 |
| GB2D5 | 2.2±0.29^a^ | 3.4±0.36^b^ | 5±0.00^c^ | 5±0.00^c^ | - | 106.39 | 0.00 |
| AKD2 | 0.7±0.46^a^ | 5±0.00^b^ | 5±0.00^b^ | 5±0.00^b^ | - | 256.00 | 0.00 |
| ATD1 | 0.7±0.68a | 4.2±0.29b | 5±0.00b | 5±0.00b | - | 90.71 | 0.00 |
| ATD2 | 2.3±0.40a | 4.2±0.76b | 5±0.00b | 5±0.00b | - | 27.37 | 0.00 |
| GK1D5 | 1.7±0.76a | 4.4±0.53b | 4.8±0.29b | 4.5±0.50b | - | 21.59 | 0.00 |
| BTD1 | 2.9±0.36a | 3.7±0.29b | 5±0.00c | 5±0.00c | - | 60.77 | 0.00 |
| BTD4 | 2.5±1.32a | 4.1±1.02a | 4.6±0.75a | 4.7±0.58a | - | 3.27 | 0.08 |
| BTjD1 | 3.8±0.29a | 4.3±0.64b | 5±0.00b | 5±0.00b | - | 8.03 | 0.01 |
| BTjD2 | 3.7±0.76a | 4.8±0.29b | 5±0.00b | 5±0.00b | - | 7.46 | 0.01 |
| BTrD1 | 3.4 ±0.36a | 4.6±0.40b | 5±0.00b | 5±0.00b | - | 23.38 | 0.00 |
| BTrD3 | 2.2±0.76a | 3.5±0.50b | 5±0.00c | 5±0.00c | - | 26.80 | 0.00 |
| NMTD2 | 2.5±0.50a | 3.8±0.29b | 4.8±0.29b | 4.6±0.51b | - | 19.27 | 0.00 |
| NMTD5 | 4±0.50a | 4.9±1.73b | 5±0.00b | 5±0.00b | - | 10.11 | 0.00 |
| NMTjD3 | 2.7±0.58a | 3.8±0.29b | 5±0.00c | 4.5±0.50c | - | 18.33 | 0.00 |
| NMTjD4 | 3.7±0.29a | 4.7±0.58b | 5±0.00b | 4.1±0.51a | - | 6.29 | 0.02 |
| NMKD2 | 3.7±0.57a | 4.2±0.76a | 5±0.00b | 5±0.00b | - | 5.73 | 0.02 |
| MABD1 | 4.1 ±0.53a | 4.7±0.58a | 5±0.00a | 5±0.00a | - | 3.53 | 0.07 |
| MABD3 | 3.3±0.58a | 4.2±0.76a | 4.9±0.17a | 4.3±0.76a | - | 3.29 | 0.08 |
| RTj1D5 | 2±1.00a | 4.2±0.76b | 5±0.00b | 5±0.00b | - | 15.21 | 0.00 |
| RTj2D2 | 3±0.00a | 3.9±0.36b | 4.5±0.87b | 3.4±0.36b | - | 4.99 | 0.03 |
| RTj2D4 | 2.6±0.51a | 3±0.50a | 4.5±0.44b | 3±1.00a | - | 5.06 | 0.03 |
| RTj3D3 | 2.8±.36a | 3.7±0.25b | 5±0.00c | 5±0.00c | - | 71.17 | 0.00 |
| DBTj1D1 | 0.5±0.50a | 1.5±0.50a | 3.7±0.29b | 3.2±0.29b | - | 38.79 | 0.00 |
| DMTD2 | 2.7±0.40a | 4±0.50b | 5±0.00c | 5±0.00c | - | 32.65 | 0.00 |
| DMTjD1 | 2.7±0.58a | 4.7±0.46b | 5±0.00b | 5±0.00b | - | 27.99 | 0.00 |
| DGTD2 | 3.3±0.40a | 4.4±0.60b | 5±0.00b | 5±0.00b | - | 15.21 | 0.00 |
| DGTjD1  AMBD2 | 3.4±0.36a  2.7±0.8 | 4.3±0.29b  3.5±0.6 | 5±0.00c  5.0±0.0 | 5±0.00c  5.0±0.0 | -  - | 32.25  16.29 | 0.00  0.00 |

**Key:** Mean values superscripted with different letter across the raw are significantly different at p- value 0.05

(-) denotes no fermentation

**Table 6.** Dry weight of isolates grown at different temperature after 72h of incubation

| **Isolate** | **Incubation Temperature in ^0^C** | | | | | **ANOVA** | |
| --- | --- | --- | --- | --- | --- | --- | --- |
|  | **20** | **25** | **30** | **35** | **40** | **F value** | **P value** |
| GT1D1 | 64.3±1.5^b^ | 82.7±3.1^c^ | 89.3±6.8^c^ | 62±7.0^b^ | 9.7±2.1^a^ | 131.9 | 0.0 |
| GT1D2 | 60±5.3^b^ | 72±11.3^c^ | 76±2.0^c^ | 59.7±6.8^b^ | 12.7±2.5^a^ | 45.4 | 0.00 |
| GT1D3 | 57.3±7.6^b^ | 70.7±4.0^c^ | 85±2.6^d^ | 59.7±2.5^b^ | 9.0±2.0^a^ | 135.2 | 0.00 |
| GT1D4 | 64.3±9.3^b^ | 79.3±6.1^c^ | 81.3±8.1^c^ | 64±8.7^b^ | 10.7±2.1^a^ | 45.9 | 0.00 |
| GT3D2 | 77±3.6^c^ | 82.3±5.7^c^ | 90±6.2^c^ | 70.3±5.68^b^ | 11.7±1.5^a^ | 124.0 | 0.00 |
| GTj1D2 | 63.3±8.3^b^ | 88±9.2^c^ | 91±7.8^c^ | 65.7±11.1^b^ | 8.3±2.5^a^ | 48.2 | 0.00 |
| GTj1D3 | 76.7±3.0^c^ | 79±3.6^c^ | 84.7±5.0^c^ | 69.3±7.0^b^ | 11.7±3.2^a^ | 125.1 | 0.00 |
| GTj2D2 | 58.7±4.2^b^ | 67±12.3^b^ | 84±12.8^c^ | 66.7±6.1^b^ | 13.0±3.0^a^ | 28.7 | 0.00 |
| GTj2D3 | 55.3±4.2^b^ | 72±2.0^d^ | 77.3±3.0^d^ | 66.7±3.5^c^ | 7.7±1.5^a^ | 261.5 | 0.00 |
| GTj3D1 | 54.7±5.7^b^ | 70.7±2.1^c^ | 75±1.0^c^ | 58±3.6^b^ | 5.6±1.5^a^ | 216.8 | 0.00 |
| GTj3D2 | 49.3 ±6.7^b^ | 55.3±5.7^c^ | 67.3±2.5^c^ | 49.3±2.5^b^ | 10.3±1.5^a^ | 75.1 | 0.00 |
| GTj3D3 | 71.3±3.1^c^ | 76.3±1.5^c^ | 82.3±1.5^c^ | 67±3.0^b^ | 10.7±2.5^a^ | 430.2 | 0.00 |
| GTj3D5 | 50.7±3.1^b^ | 61±1.0^c^ | 78±2.0^d^ | 57±3.5^c^ | 10.0±2.0^a^ | 312.2 | 0.00 |
| GB2D3 | 70 ±2.0^b^ | 77±3.0^c^ | 80.3±2.5^c^ | 68±3.0^b^ | 8.3±2.5^a^ | 382.2 | 0.00 |
| GB2D5 | 82.3±2.5^c^ | 90.3±2.1d | 92.3±5.7d | 69±1.0b | 10.3±1.5^a^ | 373.8 | 0.00 |
| AKD2 | 76.3±3.1^b^ | 80.7±2.3^c^ | 86.7±3.5^c^ | 71.7±2.5^b^ | 13.3±1.5^a^ | 373.7 | 0.00 |
| ATD1 | 76.7±5.0^c^ | 82.7±6.4^c^ | 86.7±7.2^c^ | 61.7±2.5^b^ | 12.0±2.0^a^ | 108.2 | 0.00 |
| ATD2 | 56.3 ±6.5^b^ | 68.3±7.6^c^ | 77.3±4.5^c^ | 65.3±8.5^c^ | 13.0±2.0^a^ | 48.3 | 0.00 |
| GK1D5 | 79.3±3.5^b^ | 83.3±3.5^c^ | 89.3±3.1^b^ | 75.7±3.5^b^ | 11.3±2.1^a^ | 302.6 | 0.00 |
| BTD1 | 70±6.0^b^ | 82.7±3.8^c^ | 84.3±4.5^c^ | 63±7.9^b^ | 12.7±1.5^a^ | 94.4 | 0.00 |
| BTD4 | 85±2.6^b^ | 90.7±5.0^c^ | 95.3±4.5^c^ | 60±2.0^b^ | 9.7±2.1^a^ | 30.4 | 0.00 |
| BTjD1 | 81.3±4.2^c^ | 89.7±2.5^c^ | 91.3±8.1^c^ | 61.3±2.3^b^ | 10.0±2.6^a^ | 169.9 | 0.00 |
| BTjD2 | 79.7±6.4^c^ | 81±3.6^c^ | 87±4.6^c^ | 64.3±9.3^b^ | 9.3±2.3^a^ | 91.5 | 0.00 |
| BTrD1 | 82±2.0^c^ | 86.7±2.1^c^ | 93.3±8.3^c^ | 70.7±4.0^b^ | 12.0±1.0^a^ | 170.7 | 0.00 |
| BTrD3 | 79.3±3.1^b^ | 86±5.3^d^ | 88.7±2.3^d^ | 60.7±2.1^a^ | 9.3±1.2^a^ | 335.5 | 0.00 |
| NMTD2 | 82.3±3.2^c^ | 87.7±2.1^d^ | 90.7±1.2^d^ | 70.7±2.1^b^ | 10.7±1.2^a^ | 761.4 | 0.00 |
| NMTD5 | 79.7±5.9^a^ | 84±2.7^a^ | 92.7±3.0^c^ | 70.3±2.5^b^ | 13.0±1.0^a^ | 260.6 | 0.00 |
| NMTjD3 | 69.3±2.1^b^ | 81±6.1^c^ | 84.3±6.7^c^ | 57.7±3.2^b^ | 8.7±1.2^a^ | 144.9 | 0.00 |
| NMTjD4 | 84.3±2.1^c^ | 88.7±4.2^c^ | 89.3±5.7^c^ | 48.3±6.0^b^ | 13.3±1.2^a^ | 183.0 | 0.00 |
| NMKD2 | 74.3±2.1^c^ | 82.7±2.5^d^ | 88±1.7^d^ | 60±3.0^b^ | 9.7±1.5^a^ | 599.0 | 0.00 |
| MABD1 | 76.3±2.5^c^ | 81.7±1.5^c^ | 87.3±3.2^d^ | 61.7±3.5^b^ | 15.0±1.7^a^ | 372.9 | 0.00 |
| MABD3 | 61.7±2.5^b^ | 73.7±3.5^c^ | 78±5.6^c^ | 60±5.6^b^ | 11.0±2.0^a^ | 126.9 | 0.00 |
| RTj1D5 | 82.3±3.5^c^ | 87.3±2.5^c^ | 89.3±2.1^c^ | 63.7±3.1^b^ | 9.3±1.5^a^ | 484.8 | 0.00 |
| RTj2D2 | 70.3±2.5^b^ | 82.7±3.1^c^ | 83±3.6^c^ | 60.3±3.5^b^ | 12.3±2.1^a^ | 281.8 | 0.00 |
| RTj2D4 | 72.7±4.6^b^ | 87.3±2.5^c^ | 92.7±6.4^c^ | 63.3±3.1^b^ | 14.0±2.6^a^ | 172.3 | 0.00 |
| DBTj1D1 | 49.3±7.0^b^ | 65.7±3.8^c^ | 70.7±5.1^c^ | 46.3±4.5^b^ | 6.7±1.5^a^ | 84.5 | 0.00 |
| DGTD2 | 75.7±2.1^b^ | 80.3±1.5^c^ | 83.7±2.5^c^ | 69.7±2.5^b^ | 14.0±1.0^a^ | 612.2 | 0.00 |
| DGTjD1 | 77.3±2.5^c^ | 81±1.73^c^ | 83.3±4.0^c^ | 68±3.0^b^ | 14.0±2.0^a^ | 325.3 | 0.00 |
| AMBD2 | 81.3±2.5^c^ | 86±4.0^c^ | 88.7±3.0^c^ | 73±2.0^b^ | 15.0±2.0^a^ | 355.4 | 0.00 |

**Key:** Mean values superscripted with different letter across the raw are significantly different


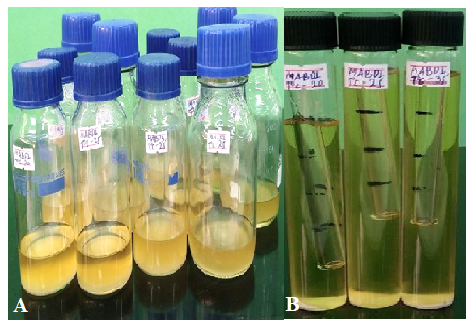


(B)

**Fig. 6** Typical picture for growth (A) and fermentation performance of isolate at different incubation temperatures (B)

**Table 7.** Growth and dry weight of isolates of at different osmostressing glucose concentration after 72 hrs of incubation at 30 ^O^C.

| Isolate | Carbohydrate concentration in Percentage | | | ANOVA | |
| --- | --- | --- | --- | --- | --- |
|  | 60 | 70 | 80 | F value | P value |
| GT1D1 | 4.2±0.76^a^ | 2.3±0.58^b^ | 1±0.50^b^ | 19.5 | 0.00 |
| GT1D2 | 4.7±0.58^a^ | 2.7±0.58^b^ | 1.1±0.12^c^ | 43.04 | 0.00 |
| GT1D3 | 6±1.00^a^ | 2.4±0.60^b^ | 1.8±0.29^b^ | 31.57 | 0.00 |
| GT1D4 | 5±1.00^a^ | 2.8±0.76^b^ | 0.9±0.12^c^ | 23.34 | 0.00 |
| GT3D2 | 3.7±0.58^a^ | 2.2±0.29^b^ | 1.1±0.17^c^ | 33.5 | 0.00 |
| GTj1D2 | 5.7±0.58^a^ | 3±1.00^b^ | 2.2±0.76^b^ | 15.67 | 0.00 |
| GTj1D3 | 5.3±0.58^a^ | 2.5±0.50^b^ | 0.9±0.12^c^ | 75.02 | 0.00 |
| GTj2D2 | 5±1.00^a^ | 2.0±0.58^b^ | 1.1±0.23^b^ | 34.87 | 0.00 |
| GTj3D3 | 5.3±0.58^a^ | 3.2±0.29^b^ | 1.2±0.29^c^ | 78.17 | 0.00 |
| GB2D5 | 6±1.00^a^ | 2.7±0.58^b^ | 1.2±0.40^b^ | 35.97 | 0.00 |
| AKD2 | 5.3±0.58^a^ | 3±0.50^b^ | 1.7±0.58^b^ | 33.82 | 0.00 |
| ATD1 | 5±1.00^a^ | 2.7±0.58^b^ | 1.8±0.29^b^ | 17.12 | 0.00 |
| ATD2 | 6.3±0.58^a^ | 3.5±0.50^b^ | 1.6±0.51^c^ | 61.10 | 0.00 |
| GK1D5 | 5.2±0.29^a^ | 2.2±0.29^b^ | 1.1±0.12^c^ | 225.17 | 0.00 |
| BTD1 | 8.1±1.00^a^ | 4.2±0.76^b^ | 2.7±0.28^b^ | 36.23 | 0.00 |
| BTD4 | 6±1.00^a^ | 3.2±1.04^b^ | 2±1.00^b^ | 12.35 | 0.00 |
| BTjD2 | 5.7±0.58^a^ | 3.3±0.58^b^ | 1.4±0.53^c^ | 43.39 | 0.00 |
| BTrD1 | 7±1.00^a^ | 3.7±0.64^b^ | 1.6±0.51^c^ | 40.16 | 0.00 |
| BTrD3 | 6.3±1.15^a^ | 3.3±1.53^b^ | 0.9±0.12^b^ | 17.90 | 0.00 |
| NMTD2 | 4.3±0.58^a^ | 2.3±0.58^b^ | 1.3±0.58^b^ | 21.00 | 0.00 |
| NMTD5 | 5.3±0.58^a^ | 2.7±0.58^b^ | 1.2±0.29^c^ | 53.44 | 0.00 |
| NMTjD3 | 5.3±0.58^a^ | 2.3±0.58^b^ | 1.7±0.58^b^ | 34.33 | 0.00 |
| NMTjD4 | 6.3±0.58^a^ | 4.3±0.58^b^ | 2.3±0.58^c^ | 36.00 | 0.00 |
| MABD1 | 7.3±1.53^a^ | 3.3±0.58^b^ | 1.5±0.50^b^ | 27.46 | 0.00 |
| MABD3 | 5.3 ±0.58^a^ | 1.8±0.29^b^ | 0.9±0.10^c^ | 115.23 | 0.00 |
| RTj2D2 | 8±1.00^a^ | 4.7±0.58^b^ | 2.3±0.58^c^ | 43.80 | 0.00 |
| DGTjD1 | 8.2±0.68^a^ | 4.2±0.76^b^ | 1.9±0.23^c^ | 5.04 | 0.00 |
| AMBD2 | 7±1.00^a^ | 3.5±1.50^b^ | 1.6±0.60^b^ | 18.49 | 0.00 |


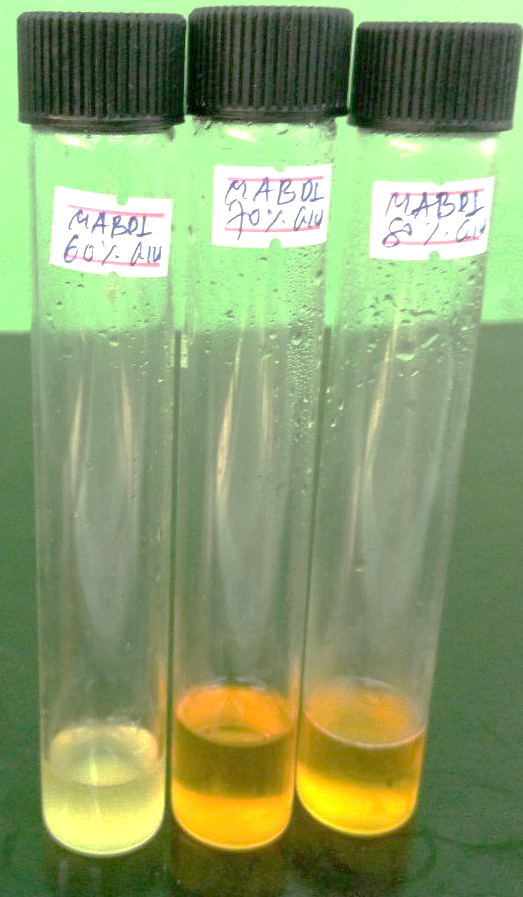


**Fig. 7** Typical picture for osmotolerance of isolate growing at higher glucose concentration

**Table 8.** Ethanol tolerance and survival of selected isolates

| Isolate | Tolerance to different ethanol concentrations | | | | | |  | | |
| --- | --- | --- | --- | --- | --- | --- | --- | --- | --- |
|  | Glucose fermentation | | | Growth (in terms of dry weight) | | |  |  |  |
|  | **10%** | **15%** | **20%** | **10%** | **15%** | **20%** | **10%** | **15%** | **20%** |
| GT1D1 | 1.2±1.04 | - | - | 66.7±4.51 | - | - | 16±5.0 | - | - |
| GT1D2 | 1.5±0.50 | - | - | 72.7±4.51 | - | - | 21.7±6.0 | - | - |
| GT1D3 | 2±0.00 | - | - | 81.0±3.00 | - | - | 41.6±8.5 | 6.1±3.9 | 1.6±1.4 |
| GT1D4 | 2±0.43 | - | - | 75.3±4.04 | - | - | 50.3±2.8 | 5.9±3.4 | - |
| GT3D2 | 2.5±0.50 | - | - | 77.0±2.65 | - | - | 57.1±4.3 | 9±0.8 | 3.2±1.1 |
| GTj1D2 | 1.5±0.40 | - | - | 70.7±1.15 | - | - | 17.7 ±4.3 | - | - |
| GTj1D3 | 1.8±0.25 | - | - | 79.3±3.51 | - | - | 50.5±7.4 | 7.4±2.1 | - |
| GTj2D2 | 1.9±0.96 | - | - | 75.3±3.21 | - | - | 65.6±6.9 | 11.5±6.9 | 2.8±1.0 |
| GTj3D3 | 1.8±0.29 | - | - | 76.0±4.58 | - | - | 44.3±10.8 | 3.7±2.1 | - |
| AKD2 | 3.5±0.50 | - | - | 81.3±2.51 | - | - | 56.5±11.0 | 5±2.1 | 0.3±0.6 |
| ATD1 | 2.1±0.81 | - | - | 73.7±2.51 | - | - | 20.5±3.5 | - | - |
| ATD2 | 3.4±0.36 | - | - | 78.0±1.73 | - | - | 40.7±8.3 | 4.2±0.9 | - |
| GK1D5 | 2.3±1.15 | - | - | 76.3±1.53 | - | - | 57.7±1.8 | 8.7±4.0 | 1.8±0.8 |
| BTD1 | 4.0±0.50 | - | - | 76.7±3.78 | - | - | 56.6±11.0 | - | - |
| BTD4 | 1.1±0.40 | - | - | 60.7±4.04 | - | - | 18.7±3.5 | - | - |
| BTjD1 | 3.2±0.76 | - | - | 77.3±3.05 | - | - | 24.4±3.0 | - | - |
| BTjD2 | 1.6±0.60 | - | - | 71.0±1.73 | - | - | 17.6±1.6 | - | - |
| BTrD1 | 2±0.50 | - | - | 78.0±3.00 | - | - | 31.2±11.3 | 9.5±0.3 |  |
| BTrD3 | 0.2±0.29 | - | - | 70.0±3.00 | - | - | 22.9±2.6 | - | - |
| NMTD2 | 1.7±0.25 | - | - | 69.3±2.51 | - | - | 14.5±2.7 | - | - |
| NMTD5 | 2.6±0.51 | - | - | 78.0±2.64 | - | - | 21.7±5.0 | - | - |
| NMTjD3 | 1.1±0.36 | - | - | 67.7±3.21 | - | - | 19. 3±2.8 | - | - |
| NMTjD4 | 2.3±0.26 | - | - | 76.3±2.51 | - | - | 27.3±6.1 | - | - |
| MABD1 | 2.1±0.17 | - | - | 79.0±1.73 | - | - | 37.8±8.3 | 4.7±2.8 | - |
| MABD3 | 0.8±0.76 | - | - | 76.0±4.36 | - | - | 25±2.6 | - | - |
| RTj2D4 | 1.5±0.00 | - | - | 75.0±2.00 | - | - | 32.5±8.9 | - | - |
| RTj2D2 | 2.8±1.10 | - | - | 75.7±5.13 | - | - | 29.1±5.7 | - | - |
| DGTD2 | 1.6±0.32 | - | - | 72.3±7.02 | - | - | 22.3±1.3 | - | - |
| DGTjD1 | 3.5±0.25 | - | - | 76.7±6.11 | - | - | 31.9±10.8 | 8±6.4 | - |
| AMBD2 | 3.3±0.26 | - | - | 76.0±4.00 | - | - | 39.2±5.5 | 4.5±2.7 | - |

**Key:** (-) denotes not ferment, grow and survive


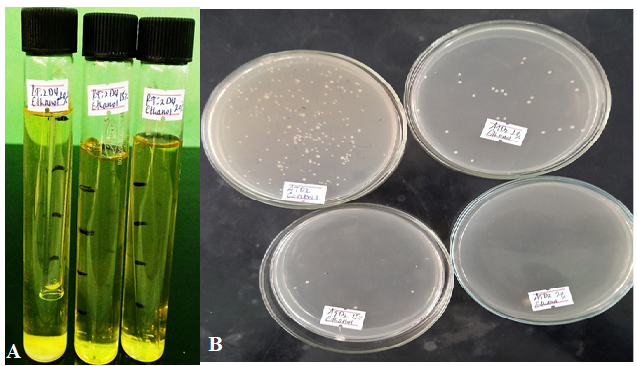


**Fig. 8** Picture for ethanol tolerance and survival assessment


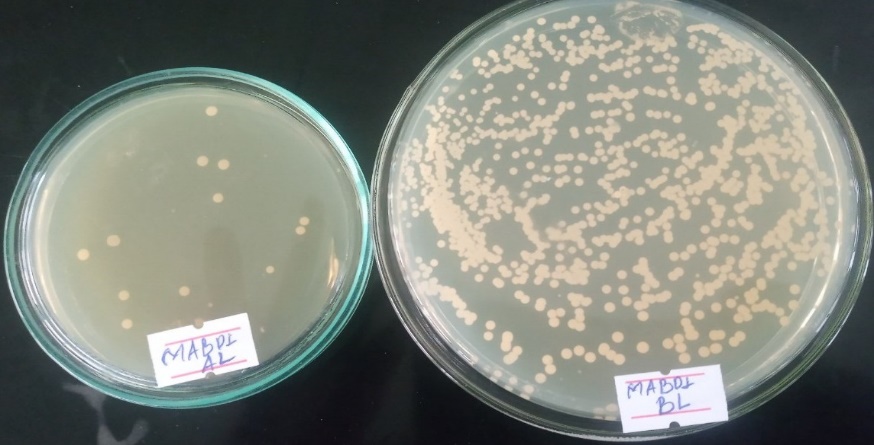


**Fig. 9** Typical picture flocculation percentage determination


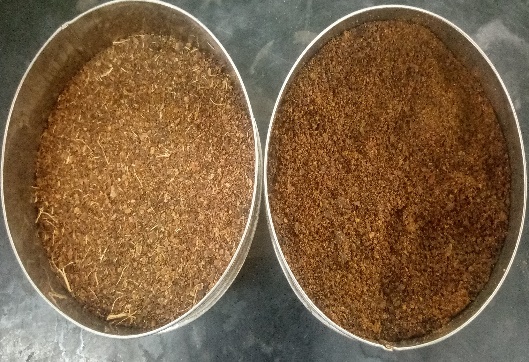

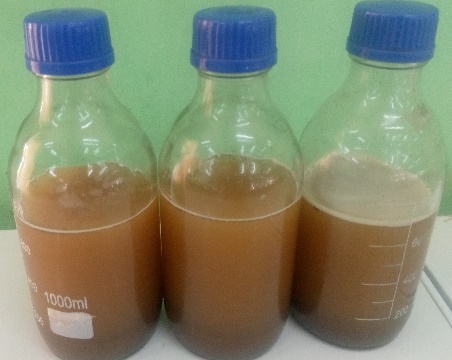

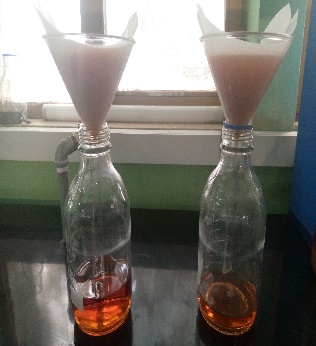

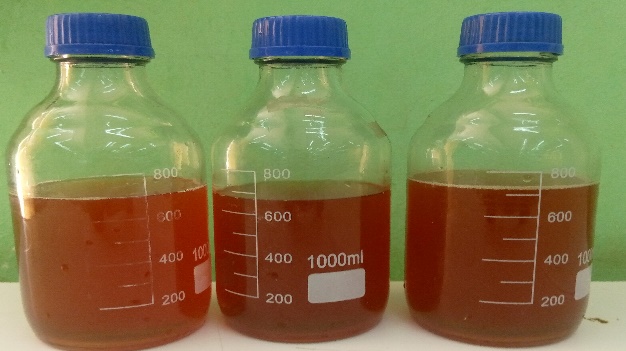


**(D)**

**(C)**

**(B)**

**(A)**

**Fig. 10** Unsieved and sieved banana peel powder **(A),** Banana peel powder macerated with distilled water for steam pretreatment **(B**), filtration of banana peel hydrolysate using Whatman no.1 filter paper **(C)** and banana peel hydrolysate used
for ethanol production **(D)**


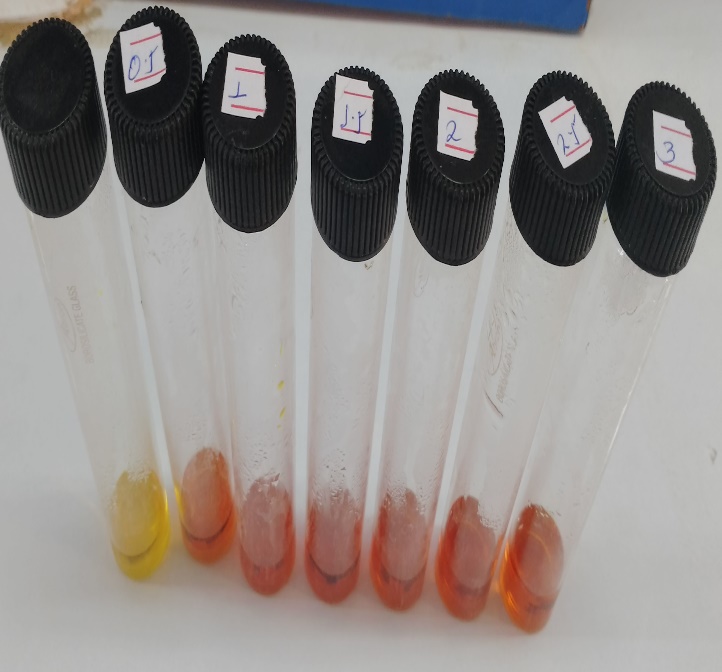


**(B)**

**(A)**

**Fig 11** Reduced sugar determination using DNS method (A) and Glucose standard curve (B)


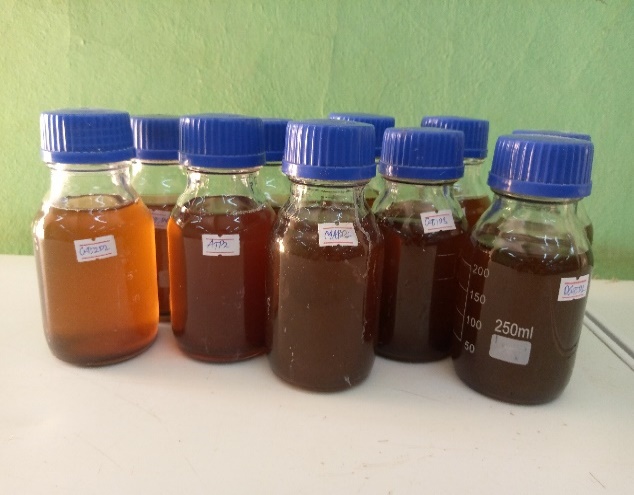

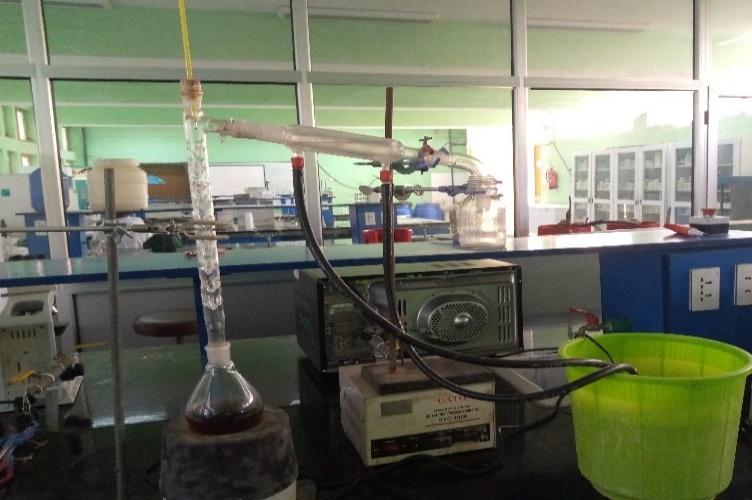


**(B)**

**(A)**

**Fig.12** Fermentation of banana peel hydrolysate **(A)** and distillation of fermented broth **(B)**

**(B)**


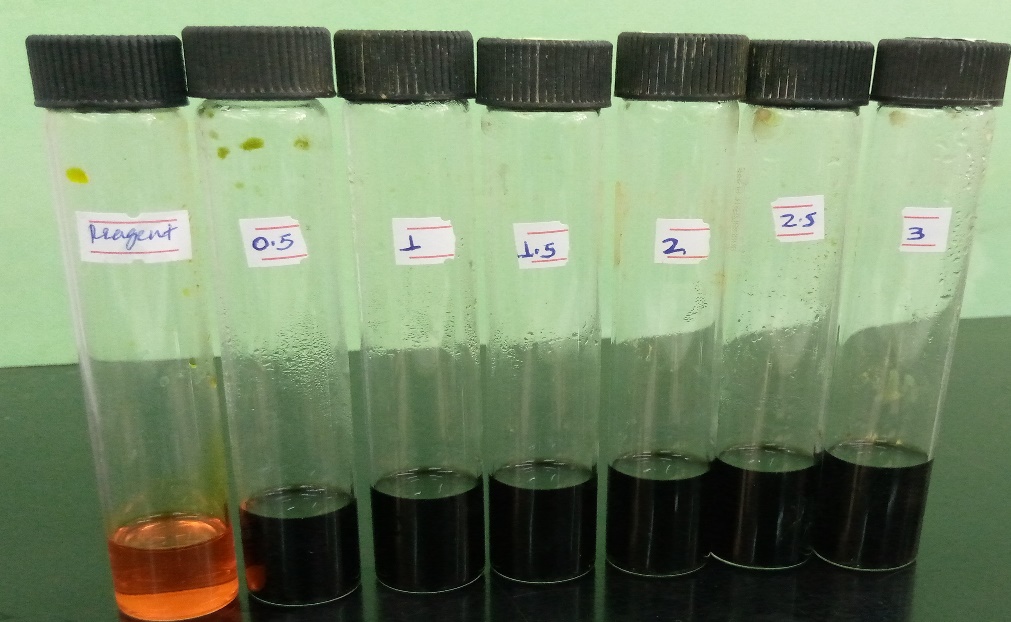
**Fig. 13** Potassium dichromate colorimetric ethanol concentration estimation using absolute ethanol (A) and ethanol standard curve (B)

**(A)**


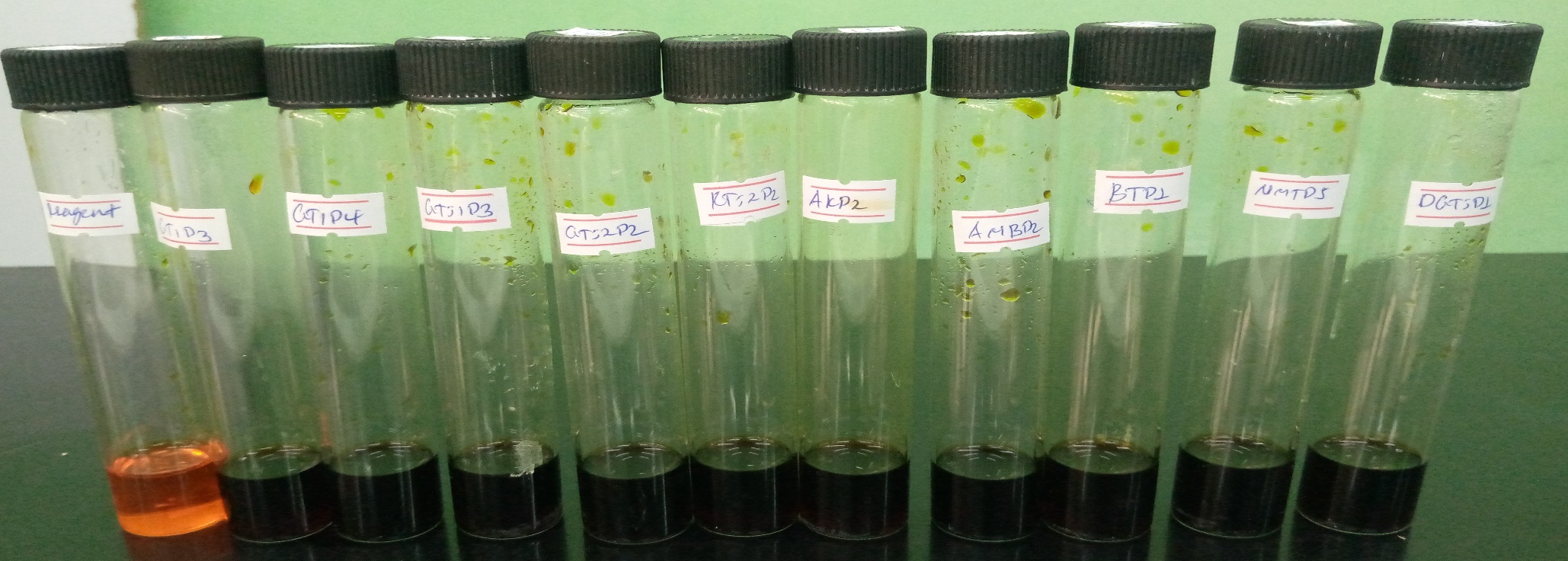


**Fig.** **14** Isolates’ ethanol yield estimation using potassium dichromate


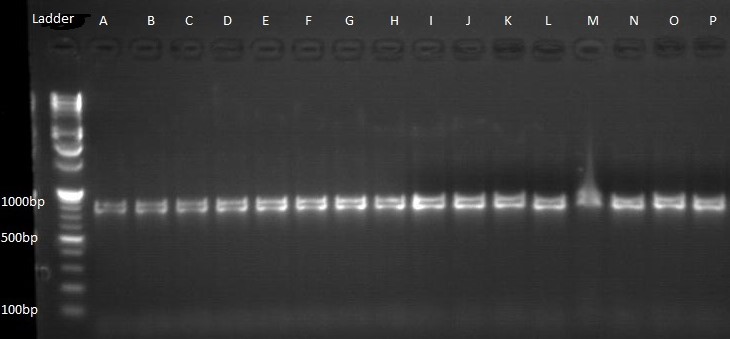


**Fig.** 15 PCR products of the ITS regions of isolates. A (GT1D3), B (GT1D4), C (GTj1D3), D (GTj2D2), E (GK1D5), F (AKD2), G (ATD2), H (BTD1), I (BTrD1), J (NMTD5), K (NMTjD4), L (MABD1), M (RTj2D2), N (DGTjD1), O (AMBD2) and P (GT3D2)
